# Impact of glyphosate and its mixture with 2,4-D and dicamba on gut biochemical function, intestinal barrier integrity and microbiome composition in adult rats with prenatal commencement of exposure

**DOI:** 10.1007/s00204-026-04409-9

**Published:** 2026-04-28

**Authors:** Robin Mesnage, Scarlett Ferguson, Paraskevi-Maria Nechalioti, Liliana Cercelaru, Mohamad Alaa Hbous, Anca Oana Docea, Aristidis Tsatsakis, Demetrios Kouretas, Michael N Antoniou

**Affiliations:** 1 Gene Expression and Therapy Group, Faculty of Life Sciences and Medicine, Department of Medical and Molecular Genetics, King’s College London, Guy’s Hospital, London, SE1 9RT UK; 2 Department of Biochemistry and Biotechnology, University of Thessaly, Viopolis, Mezourlo, 41500 Larissa Greece; 3 Department of Anatomy and Embryology, University of Medicine and Pharmacy of Craiova, Craiova, 200349 Romania; 4 Department of Toxicology, University of Medicine and Pharmacy of Craiova, Craiova, 200349 Romania; 5 Department of Forensic Sciences and Toxicology, Faculty of Medicine, University of Crete, 71003 Heraklion, Greece; 6 Present Address: Buchinger Wilhelmi Clinic, Wilhelmi-Beck-Straße 27, 88662 Überlingen, Germany

**Keywords:** Glyphosate, Dicamba, 2,4-D, Gut microbiome, Gut dysbiosis, Intestinal barrier integrity

## Abstract

Intensive use of glyphosate-based herbicides in genetically modified (GM) and non-GM agriculture has resulted in widespread glyphosate-resistant weeds. In response, the agricultural biotechnology industry has launched GM crops tolerant to glyphosate plus 2,4-D and glyphosate plus dicamba. Consequently, people are increasingly exposed to mixtures of these herbicides, yet no studies have been conducted to assess health risks. Intestinal structure and integrity, as well as gut microbiome composition and function, are recognised contributors to disease. Therefore, we investigated the effects of glyphosate alone and in combination with 2,4-D and dicamba on gut structure and function. Pregnant Wistar rats were administered via drinking water from gestation day 6 with glyphosate at the European Union (EU) no observed adverse effect level (NOAEL: 50 mg/kg bw/day) and acceptable daily intake (ADI: 0.5 mg/kg bw/day), and with an ADI mixture of glyphosate (0.5 mg/kg bw/day), 2,4-D (0.02 mg/kg bw/day) and dicamba (0.3 mg/kg bw/day). Offspring continued this regimen for 13 weeks post-weaning. Large and small intestinal tissues and gut content were isolated and analysed for inflammation, gut epithelial integrity, oxidative stress, microbiota composition and histopathology. The glyphosate NOAEL and, to a greater degree, the glyphosate, dicamba, 2,4-D mixture resulted in increased gut inflammation and permeability, associated with oxidative stress and altered microbial composition. Histological analysis confirmed structural alterations and inflammation in large and small intestine. Effects were more pronounced in large intestine and females. Our results identify exposure to glyphosate alone and a mixture of glyphosate, 2,4-D and dicamba as risk factors for gut structure and function dysbiosis.

**Supplementary Information:**

The online version contains supplementary material available at 10.1007/s00204-026-04409-9.

## Introduction

The potential negative consequences from the extensive use of herbicides in chemical-based, intensive agriculture is of increasing concern (Mesnage and Zaller 2021). Among the commonly employed herbicides, glyphosate, the declared “active substance” in commercial formulations such as Roundup^®^, has received significant attention due to its large-scale global application and potential environmental (Klátyik et al. 2023, 2024, 2025) and health impacts (Myers et al. 2016; Van Bruggen et al. 2028, Peillex and Pelletier 2020) including potential to induce fatty liver disease (Riechelmann-Casarin et al. 2025), neurological defects (Costas-Ferreira et al. 2022) and cancer (Weisenburger 2021; Weisenburger 2026 ; Panzacchi et al. 2025). Glyphosate-based herbicide (GBH) use increased exponentially from 1996 with the launch of glyphosate-tolerant genetically modified (GM) crops (soybeans, maize, cotton, canola, sugar beet, alfalfa, etc.), particularly in North and South America. Estimates of global glyphosate active ingredient use stand at approximately 749 million kg annually (Benbrook 2016; Maggi et al. 2020). However, overreliance on glyphosate as a weed control agent in GM and non-GM (e.g. pre-harvest desiccation on cereals) cropping systems has inevitably led to the emergence of glyphosate resistant weeds. A total of 48 weed species have evolved glyphosate resistance worldwide (Baek et al. 2021) with greatest infestation in GM crop fields in the United States (Heap and Duke 2018). As a result, farmers use combinations of herbicides such as glyphosate along with 2,4-dichlorophenoxyacetic acid (2,4-D) and dicamba to improve effectiveness of weed control. The use of glyphosate with other herbicides has been exacerbated with the introduction of GM crops tolerant to glyphosate plus 2,4-D and glyphosate plus dicamba (Mesnage and Antoniou 2021). This has resulted in increased 2,4-D use to over 15.1 million kg in 2020, a rise of nearly 200% over 2002 levels (Freisthler et al. 2022; Survey U.G. 2021a) and annual use of dicamba increasing from approximately 3.6 million kg in 2016 to over 14 million kg by 2019 (Survey U.G. 2021b) in US agriculture. This raises the concern that people will be exposed to increasing amounts of a mixture of glyphosate, 2,4-D and dicamba. This possibility was confirmed in a biomonitoring investigation, which compared urine levels of 2,4-D and dicamba in pregnant women before (2010−1012) and after (2020–2022) the introduction of glyphosate plus 2,4-D- and glyphosate plus dicamba-tolerant GM crops (Daggy et al. 2024). The frequency of detection in urine of dicamba increased from 28% in the 2010–2012 cohort to 70% in the 2020–2022 group with a significant increase (greater than 4-fold) between the two cohorts in urinary geometric mean concentration. All samples analysed also contained 2,4-D, with an increase in the geometric mean levels detected between the 2010–2012 and 2020–2022 cohorts of women, but this did not reach statistical significance (Daggy et al. 2024). Health risks from exposure to a mixture of glyphosate, 2,4-D and dicamba are currently unknown and thus represent a knowledge gap with potential public health implications.

One physiological system that can be impacted by herbicides is the gut, which can include effects on biochemical function, intestinal barrier integrity and the microbiome (Tsiaoussis et al. 2019). The gut microbiome plays a fundamental role in numerous physiological processes, including digestion of food, nutrient metabolism, immune regulation, and maintenance of gut intestinal barrier integrity (Camilleri 2019). Disruptions to the gut microbiome have been associated with a range of health disorders, including metabolic syndrome, inflammatory bowel diseases, autoimmunity and neurodevelopmental disorders (Gacesa et al. 2022; Ghosh et al. 2022).

While previous studies have primarily focused on evaluating the toxicity of individual herbicides, there is a critical need to investigate the effects of herbicide mixtures, which reflects real-life exposures (Martin et al. 2021). When herbicides are combined, their interactions can result in synergistic, additive, or antagonistic effects, leading to substantial alterations in toxicity compared to individual components (Docea et al. 2018; Delfosse et al. 2021; Mesnage et al. 2021a). Toxicity tests conducted for regulatory purposes primarily consider high-dose exposures to single “active ingredients” tested in isolation, overlooking the potential risks associated with chronic low-dose exposure to mixtures, as encountered in real-world situations (Myers et al. 2009; Tsatsakis et al. 2017). Assessing the effects of low-dose exposure to mixtures is essential for a comprehensive understanding of potential health consequences and the formulation of appropriate regulatory policy.

In an effort to mimic a real-world scenario with potential of enhanced toxicity arising from exposure to a mixture to glyphosate, 2,4-D and dicamba, we conducted an in vivo study using a rat model system, and compared this to exposure to glyphosate alone. In order to be aligned with government regulatory practice, we did not include an assessment of commercial formulations of these herbicide active ingredients (Mesnage and Antoniou 2018; Mesnage et al. 2019). Furthermore, again to more closely reflect a realistic human life situation, animals were exposed starting at a pre-natal stage of development (gestation day (GD) 6) until early adulthood to either glyphosate alone or a glyphosate, 2,4-D and dicamba mixture, acknowledging that the fetus constitutes a heightened window of sensitivity to toxicant insult, which can lead to developmental defects and adult onset illnesses (Rager et al. 2020; Potiris et al. 2025). Animals were evaluated to assess the impact of exposure on gut biochemical function (inflammation, redox status), integrity and microbiome (bacterial and fungal population) status. We also assessed markers of “leaky gut”, a condition characterized by increased intestinal barrier permeability, as an indicator of gastrointestinal dysfunction (Camilleri 2019), which can trigger immune responses and contribute to pathology in the gut and elsewhere in the body of the consumer (Christovich and Luo 2022; Aleman et al. 2023).

## Materials and methods

### Herbicide active ingredients

Glyphosate (≥ 98.0% purity), dicamba (≥ 98.0% purity), and 2,4-D (2,4-dichlorophenoxyacetic acid) (97.0% purity) were of analytical grade and purchased from Sigma-Aldrich, Merck KGaA, Germany. Stock solutions of 0.5 g/L glyphosate, 3 mg/L dicamba and 0.2 mg/L 2,4-D were prepared in drinking water and stored at 4 °C. Daily solutions were prepared from the stock solutions for administration in the drinking water of each group of animals.

## Animal herbicide exposure procedure

The experimental design of this investigation is described in the published study protocol (Mesnage et al. 2023). The study was conducted in accordance with the European Community Council Directive (2010/63/EU) on the use and care of animals for scientific purposes and approved by the Ethical Committee of the University of Medicine and Pharmacy Craiova, Romania. The study design was in accordance with OECD Test Guideline 414 (Prenatal Developmental Toxicity Study) and OECD Test Guideline 408 (90-day Repeated Dose Oral Toxicity Study).

Briefly, groups of pregnant Wistar rats, with 5 animals per group, were subjected to the following herbicide exposures: (i) control group receiving only standard diet and drinking water, (ii) one group given glyphosate at the EU acceptable daily intake (ADI; 0.5 mg/kg body weight (bw)/day) and another the no-observed-adverse-effect level (NOAEL; 50 mg/kg bw/day), (iii) the fourth group administered with glyphosate, 2,4-D, and dicamba with each at their respective EU ADI (0.3 mg/kg bw/day dicamba; 0.5 mg/kg bw/day glyphosate; 0.02 mg/kg bw/day 2,4-D). The substances were administered via drinking water starting from gestation day (GD) 6 until weaning (5 litters in each group) and continued with the offspring (10 male and 10 female animals per exposure group) for another 13 weeks post-weaning. Specifically, approximately two males and two females from each litter in the respective exposure group (10 males and 10 females in total per exposure group), were selected for continuous exposure for an additional 13 weeks, according to the initial exposure regimen. Animals were sacrificed by exsanguination after general anaesthesia with xylazine and ketamine at the end of the 13-week postnatal period of exposure. The contents of the large intestine caecum and small intestine ileum compartments were collected and stored at − 80 °C. Ileum and caecum tissue samples were either stored at − 80 °C or immediately fixed in 10% formalin solution for 24 h for histopathological examination.

## Tissue processing and histopathological evaluation

Histopathological analysis was conducted on samples from the small intestine (ileum) and colon (caecum). The samples were immediately fixed in 10% neutral-buffered formalin for 24 h at room temperature to preserve tissue morphology. After fixation, the tissues were washed in phosphate-buffered saline (PBS) and embedded in paraffin. Hematoxylin and eosin (H&E) staining was performed to evaluate general histopathological changes. Paraffin-embedded blocks were sectioned into 4 μm slices using a Leica RM 2125 microtome. The H&E stained tissue sections were examined under a Panthera L light microscope (Motic Europe, S.L.U., Barcelona, Spain) at various magnifications. Because the observed changes were focal rather than diffuse, we used a standardized sampling approach for semi-quantitative scoring: three predefined/random non-overlapping high-power fields (x400) were selected per slide and averaged. This approach was chosen to maintain consistency across animals and groups, and avoid disproportionate scoring driven by a single focal “hotspot” or by field selection. The morphological characterization of lesions was based on whole-slide evaluation and was performed by two experienced pathologists, who were blinded to the experimental groups. In cases of disagreement in interpretation, the pathologists discussed the findings and reached a consensus.

Lesions were identified and reported using standardized nomenclature and diagnostic criteria in accordance with the International Harmonization of Nomenclature and Diagnostic Criteria for Lesions in Rats and Mice (INHAND) recommendations for gastrointestinal tract pathology (Nolte et al. 2016). Histopathological alterations were graded using a semiquantitative severity scoring system based on the severity the lesion, extent of tissue involvement and number of animals affected (Meyerholz et al. 2018).

The scoring system for lesion severity was as follows: (−) no change: no histopathological changes observed in the tissue; (+) mild: histopathological changes are minimal and limited to small areas of the tissue; (++) moderate: histopathological changes are more pronounced and affect a larger area but are still somewhat limited; (+++) severe: pronounced, severe histopathological changes with widespread or very prominent changes in tissue morphology.

## RT-qPCR analysis

The expression of 5 genes indicative of alterations in the structure of tight junctions was performed using real-time quantitative polymerase chain reaction (RT-qPCR) analysis of total RNA from large intestine (caecum) and ileum. Extracted total RNA was retrotranscribed to cDNA using the SuperScript VILO cDNA Synthesis Kit (ThermoFisher Scientific, Loughborough, UK). A total of 100 ng cDNA was then amplified using TaqMan assays to determine mRNA levels of Muc2 (Rn01498206_m1), Ocln (Rn00580064_m1), Zo1 (Rn07315717_m1), Cldn3 (Rn00581751_s1) and Cldn4 (Rn01196224_s1). In addition, the expression of 3 genes, namely *Il22* (Rn01760432_m1), *Lcn2* (Rn00590612_m1) and *Tlr4* (Rn01458370_m1), as markers for the occurrence of inflammation, were also assessed in both the large intestine and ileum. All RT-qPCR assessments of target mRNA levels used Gapdh (Rn01775763_g1) as an internal reference standard. Reactions were conducted as technical duplicates, with a TaqMan Fast Advanced Master Mix (ThermoFisher Scientific, UK) on the Applied Biosystems QuantStudio 6 Flex Real-Time quantitative PCR (RT-qPCR) System. The 2^− ΔΔCt^ method was used to calculate the relative gene expression of the target genes (Livak and Schmittgen 2001).

## Calprotectin ELISA

Intestinal inflammation was investigated by determining levels of calprotectin in 100 µL aliquots of caecum content using the Immundiagnostik S100A8/S100A9 ELISA kit (Immundiagnostik, Bensheim, Germany Catalog No: KR6936) in accordance with the manufacturer’s instructions. Absorbance following addition of substrate was read at 450 nm with a microplate reader (Promega, UK). Calprotectin levels are expressed as ng/mL caecum content with a detection limit of 0.076 ng/mL.

### Evaluation of serum zonulin and bacterial lipopolysaccharides

Serum zonulin was measured using the Abbexa Rat Haptoglobin Precursor/Zonulin (HP) ELISA kit (Abbexa, Cambridge, UK Catalog No: abx256353). The ELISA was performed using 100 µL of serum as per the manufacturer’s protocol. Absorbance following addition of substrate was read at 450 nm with a microplate reader (Promega, UK). Zonulin levels are expressed as ng/mL serum.

Similarly, 50 µL of serum were used to determine LPS concentration using the Abexxa Lipopolysaccharides (LPS) ELISA kit (Abexxa, Cambridge, UK Catalog No: abx150357) in accordance with the manufacturer’s instructions with absorbance at 450 nm following addition of substrate with a microplate reader (Promega, UK). LPS levels are expressed as ng/mL serum.

## Measurement of redox biomarkers

Spectrophotometric methods were employed to measure markers of redox status (reduced glutathione, hydrogen peroxide decomposition rate, total antioxidant capacity, thiobarbituric acid reactive substances, protein carbonyls) in large intestinal tissue homogenates as previously described (Nechalioti et al. 2023).

## Gut microbiota composition

Isolation of caecum and ileum content DNA and its analysis for microbiota composition was by sequence analysis of bacterial 16 S V3–V4 and fungal ITS2 regions as previously described (Mesnage et al. 2022) as follows.

A total of 5ng DNA was amplified by polymerase chain reaction (PCR) using the Roche High-Fidelity PCR System (Roche Life Science, Welwyn Garden City, United Kingdom) in a reaction volume of 10 µL. The primers for the amplification of the bacterial 16 S V3-V4 region were: ACACTGACGACATGGTTCTACACCTAC GGGNGGCWGCAG (forward) and TACGGTAGCAGAGACTT GGTCTGACTACHVGGGTATCTAATCC (reverse). The primers for fungal ITS2 region amplification were: TCTACACTCGTCGGCAGCGTC AGATGTGTATAAGAGACAGGCATCGATGAAGAACGCAGC (forward) and GTCTCGTGGGCTCGGAGATGTGTATAAG AGACAGTCCTCCGCTTATTGATATGC (reverse).

The PCR reaction mixture included 2µL of 10X FastStart High Fidelity Reaction buffer, 0.2µL of 10µM forward or reverse primers, 0.2µL of 10mM PCR Grade Nucleotide Mix, 0.25µL platinum SuperFi II DNA Polymerase at 2U/µL and 5.35µL RNase-free water. This was added to 2µL of sample DNA at 5ng/µL and PCR amplified for 35 cycles at 95 °C for 30s, 55 °C for 30s, 72 °C for 30s, and a final extension at 72 °C for 5 min. The size of amplified products was verified by electrophoresis on a 2% agarose gel. Then, 1µL of a 100 times diluted PCR product in TE buffer (10mM Tris-HCl, 1mM EDTA, pH7.5) was used in a second round of PCR to add TSP FLD barcodes and Illumina adaptors onto PCR products. The barcoding reaction mix included 1µL of 10X FastStart High Fidelity Reaction buffer, 1.8µL of 25mM MgCl_2_, 0.5µL of DMSO, 0.2µL of 10mM PCR Grade Nucleotide Mix, 0.1µL of 5U/µL FastStart High Fidelity Enzyme Blend and 1.4µL of nuclease-free water. This was added to 4µL of Fluidigm Barcode and 1µL of the 1:100 harvested PCR product.

PCR amplification was done for 15 cycles at 95 °C for 15s, 60 °C for 30s, 72 °C for 60s, and a final extension at 72 °C for 3 min. Barcode attachment was controlled using the Agilent Tapestation 4200 instrument (Agilent, CA, United States). An equal volume of each barcoded PCR product was pooled, and the final mixture diluted to 4 nm. The pooled library was loaded onto a 300bpx2 paired-end MiSeq (Illumina, CA, United States), as per the manufacturer’s instructions.

### Bioinformatics analysis of microbiome DNA sequence data

Amplicon sequence variants (ASVs) were processed using DADA2 (v1.16) in R (v4.0.0) with pseudo-pooling and taxonomically classified via DADA2’s naïve Bayesian classifier against the SILVA database (v138) for 16 S and UNITE ITS for fungi. Functional prediction from 16 S rRNA genes was performed with PICRUSt2 via the q2-picrust2 plugin (Douglas et al. 2020).

Cleaned reads, ASV taxonomies, and metadata were integrated in phyloseq (v1.32.0) for diversity analysis. Alpha diversity (observed species, Shannon index) was assessed with ANOVA and Tukey post-hoc tests. Beta diversity was evaluated by NMDS on Bray-Curtis dissimilarities, with significance tested by PERMANOVA (adonis, vegan v2.4-2). Taxonomy visualisation used microViz (v0.7.1) and ggplot2 (v3.3.0).

Community composition was examined across taxonomic levels; genus-level differences were tested with MaAsLin2 (v0.99.12) including sex as a covariate and in sex-stratified analyses. Data were normalized by total sum scaling, variance-stabilized with arcsine square root transformation, and multiple testing was controlled using false discovery rate (FDR) correction.

### Statistical analyses

Statistical analyses for all markers except for gut microbiota data analysis was done using two-way ANOVA using the exposed groups and sex as factors followed by a Tukey’s post hoc test for multiple comparisons among the different experimental groups and was undertaken using GraphPad Prism 8.0.1 software. Normality of residuals was evaluated by a Shapiro-Wilk test. All results are expressed as mean ± standard error of the mean (SEM), and the differences were considered significant at *p* < 0.05. Statistical analysis of histopathological data was undertaken by comparing the scores of the exposure groups to the un-exposed control animals employing a chi-square test using SPSS software (IBM, NY, USA). A p-value of less than 0.05 was considered indicative of statistical significance.

## Results

We evaluated if either glyphosate or a glyphosate, dicamba and 2,4-D mixture at regulatory permissible daily intake levels could alter not only the composition of the rat gut microbiota population but also the integrity and function of the gut, in both sexes, and in the large intestine and ileum starting at a pre-natal stage of development. Animals appeared normal with a decrease in body weight observed only in males exposed to the mixture of herbicides (*p* = 1.1 × 10^−5^) (Supplementary Fig. 1A). Water consumption was lower for the group of male rats exposed to the glyphosate NOAEL (*p* = 0.0007). In females, water consumption was lower for the groups exposed to the glyphosate ADI (*p* = 4.6 × 10^−9^) and NOAEL (*p* = 0.02) (Supplementary Fig. 1B). Food consumption between control and herbicide exposure groups was not statistically significant (Supplementary Fig. 1C).

### Gut inflammation measures

**Fig. 1:**
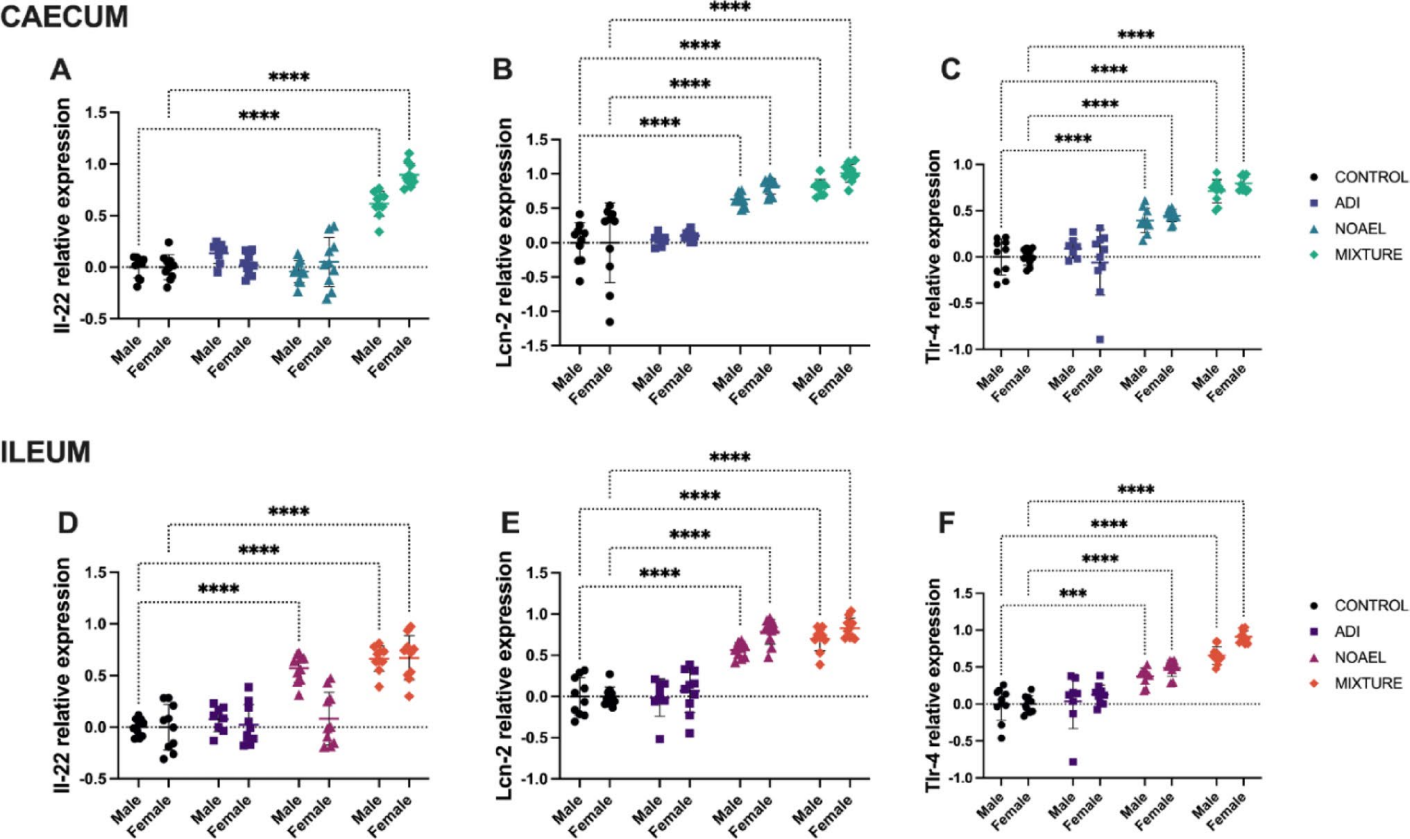
**Glyphosate NOAEL and glyphosate, dicamba, 2,4-D mixture increases expression of genes associated with intestinal inflammation.**Caecum and ileum from rats exposed to glyphosate at either its EU acceptable daily intake (ADI) or no observed adverse effect level (NOAEL) or a MIXTURE of dicamba, glyphosate and 2,4-D (at the EU ADI) from GD6 until 13 weekspost-weaning were, analysed for expression of genes associated with gut inflammation;*Il22*(**A**, caecum; **D**, ileum),*Lcn2*(**B**, caecum;**E**, Ileum) and*Tlr4*(**C**, caecum;**F**, Ileum) by RT-qPCR. Each dot represents the result from a single animal. The longer horizontal line within each dataset denotes the average value. *p < 0.05; **p < 0.01; ***p < 0.001; ****p < 0.0001 in a Tukey multiplecomparison test, which followed a one-way ANOVA.

We began by evaluating if exposure to the herbicides induced inflammation, which could lead to gut dysbiosis. First, we assessed effects on the expression of the inflammation-associated genes *Il22*, *Lcn2*, and *Tlr4* in large intestinal caecum and ileum tissue of male and female animals (Fig. 1). (Summary statistics of RT-qPCR assays in Supplementary Tables 1 and 2). In animals exposed to the glyphosate, dicamba and 2,4-D herbicide mixture, a statistically significant (*p* < 0.0001) increase compared to the untreated control groups of rats was observed in expression of all three inflammatory marker genes in large intestine (Fig. 1A–C) and ileum (Fig. 1D–F) of females and males. Average increases in expression ranged from 4.1 to 10.2-fold in the large intestine and 4.5 to 8.1-fold in the ileum (Supplementary Tables 1 and 2). Overall, there was a heightened sensitivity to exposure – that is, greater increase in gene expression in the large intestinal compartment compared to the ileum, particularly with respect to *Il22* expression (Fig. 1A). In addition, there was a significantly higher level of expression in both the ileum and large intestine in female compared to male animals treated with the herbicide mixture in all cases except for *Il22* in the ileum (Supplementary Fig. 2; Supplementary Tables 1 and 2).

Exposure to glyphosate alone led to more restricted outcomes with a statistically significant (*p* < 0.0001) average increase in expression of *Lcn2* in the large intestine (4.3- to 6.6-fold) and ileum (3.7-fold) in both female and male animals at the EU NOAEL (Fig. 1B, E; Supplementary Tables 1 and 2). We also detected a significant but more modest average increase (2.4- to 3.1-fold) in expression of *Tlr4* in the large intestine and ileum of female and male animals (Fig. 1C, F; Supplementary Tables 1 and 2). Expression of *Il22* was only found to increase (3.7-fold) in the ileum of male rats (Fig. 1D; Supplementary Table 2). Also, we observed a significantly higher expression in female rats compared to males for *Lcn2* in the ileum and large intestine in the glyphosate NOAEL groups (Supplementary Fig. 2; Supplementary Tables 1 and 2). No changes in expression of any of these inflammatory marker genes in males or females was observed at the glyphosate EU ADI (Fig. 1; Supplementary Tables 1 and 2).

**Fig. 2:**
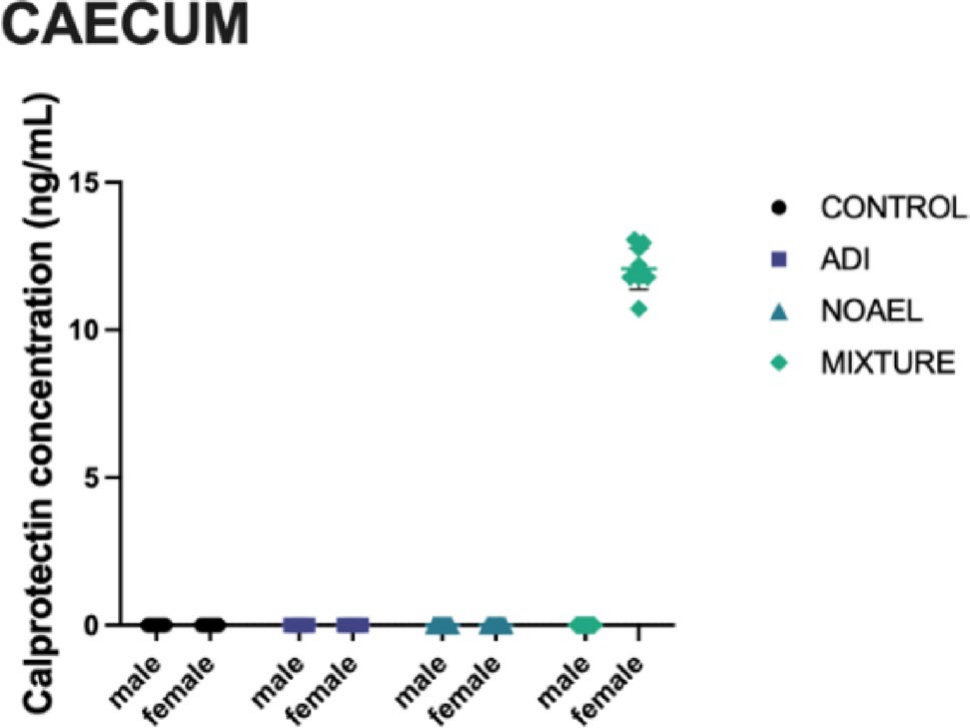
**Elevated calprotectin in caecum content reveals gut inflammation from exposure to glyphosate, 2,4-D, dicamba mixture in females.**Caecum content from rats exposed to the glyphosate acceptable daily intake (ADI) and no-observed adverse effect level (NOAEL), and ADI MIXTURE of glyphosate, 2,4-D, dicamba was analysed for calprotectin by ELISA. Control,glyphosate alone and herbicide mixture exposed male group measures were below the limit of detection. Each dot represents the result from a single animal

We next measured levels of calprotectin, a clinically established marker of gut inflammation (Jukic et al. 2021). Analysis focused on large intestinal caecum content, where we observed the greatest increases in inflammatory marker gene expression (Fig. 1; Supplementary Fig. 2; Supplementary Tables 1 and 2). Calprotectin levels could not be accurately determined in most cases as values were below the limit of detection (0.076 ng/mL), preventing statistical analysis (Fig. 2). However, exposure to the glyphosate, 2,4-D and dicamba mixture gave rise to calprotectin levels well above the limit of detection in female rats, suggesting a heightened inflammatory response. In contrast, neither male nor female rats treated with glyphosate alone at either the ADI or NOAEL showed an increase in calprotectin levels (Fig. 2).

### Gut integrity and permeability assessment

**Fig. 3:**
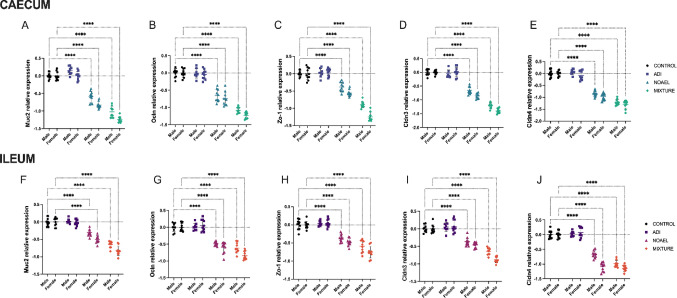
**Glyphosate NOAEL and glyphosate, dicamba, 2,4-D mixture reduces expression of tight junction protein-encoding genes.** Caecum and ileum from rats exposed to glyphosate at the acceptable daily intake (ADI) and no observed adverse effect level (NOAEL), and ADI MIXTURE of dicamba, glyphosate, 2,4-D from GD6 until 13 weeks post-weaning, was analysed forexpression of genes encoding the tight junction proteins *Muc2* ( **A**, caecum; **F**, ileum), *Ocln* ( **B**, caecum; **G**, ileum), *Zo1* ( **C**, caecum; **H**, ileum), *Cldn3* ( **D**, caecum; **I**, ileum) and *Cldn4* ( **E**, caecum; **J**, ileum) by RT-qPCR. Each dot represents the result from a single animal. The horizontal line within each dataset denotes the average value. *p < 0.05; **p < 0.01; ***p < 0.001; ****p < 0.0001in a Tukey multiple comparison test which followed a one-way ANOVA

Gut inflammation is associated with increased gut permeability (Ahmad et al. 2017). Compromised intestinal barrier integrity can lead to gut inflammation resulting in conditions such as arthritis in a rat model system (Hecquet et al. 2023), bone density loss during menopause in women (Shieh et al. 2020), obesity (Keirns et al. 2023), mental health problems (Madison and Bailey 2024) and cancer (Sánchez-Alcoholado et al. 2020). We therefore next investigated whether the herbicides affected intestinal integrity and potential permeability by measuring the expression of the tight junction protein-encoding genes *Muc2*, *Ocln*, *Zo1*, *Cldn3*, and *Cldn4* in the large intestine and ileum. Our results show that exposure to the glyphosate, dicamba, and 2,4-D mixture significantly (*p* < 0.0001) reduced expression of all five tight junction protein encoding genes in female and male animals in both the large intestine (Fig. 3A-E) and ileum (Fig. 3F-J). In females, there was a 16.7- to 25-fold average decrease in expression in the large intestine and a 5.9- to 14.3-fold average decrease in the ileum (Supplementary Table 1). In males, a 7.7- to 16.7-fold average decrease in the large intestine and a 4- to 10-fold average decrease in the ileum was observed (Supplementary Table 2).

Exposure to the glyphosate ADI did not result in a measurable change in the expression of these 5 gut integrity marker genes in female or male rats in either the large intestinal (Fig. 3A–E) or ileum (Fig. 3F–J) compartments (Supplementary Tables 1 and 2). However, at the higher glyphosate NOAEL, we observed a significant (*p* < 0.0001) decrease in expression of all 5 gut integrity marker genes in female animals in both the large intestine (Fig. 3A-E) and ileum (Fig. 3F–J). Similarly, in male rats a significant (*p* < 0.0001) decrease in expression for all but one (*Muc2*) of the gut integrity marker genes was observed (Fig. 3F–J). These decreases ranged from an average of 7.7- to 16.7-fold in the large intestine and an average of 4- to 10-fold in the ileum of females (Supplementary Table 1). In males, we observed an average 2.4- to 7.1-fold decrease in the large intestine and an average 2- to 4.8-fold in the ileum (Supplementary Table 2). Notably, the effects of glyphosate exposure at the NOAEL were more pronounced in the large intestine compared to the ileum, and in females compared to males.

A comparison in the expression of these gut integrity marker genes between male and female rats showed a modest but significantly lower (*p* < 0.05) expression of only *Cldn4* in the large intestine of females (Supplementary Fig. 3).

**Fig. 4:**
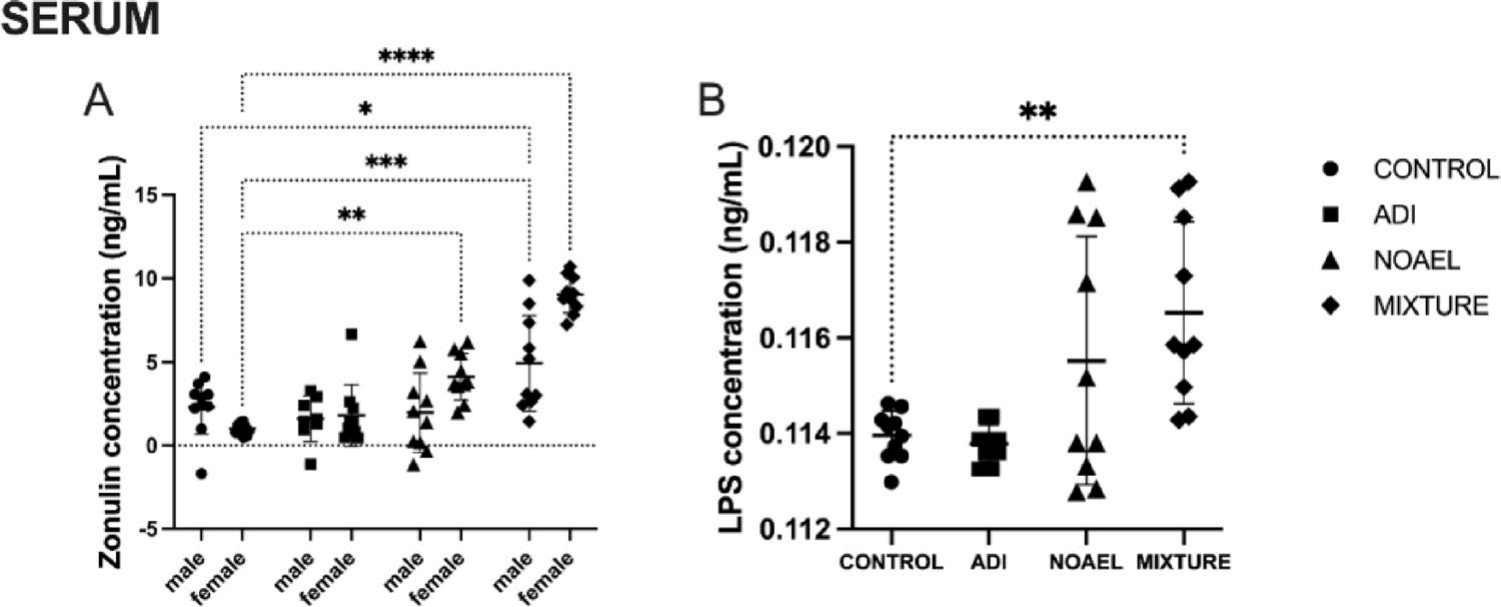
**Increased serum zonulin and bacterial lipopolysaccharides reveal compromised gut integrity from exposure to the glyphosate, 2,4-D, dicamba mixture**. Serum from rats exposed to glyphosate at the ADI and NOAEL and ADI MIXTURE of glyphosate, 2,4-D, dicamba MIXTURE was analysed for zonulin (**A**) and bacterial lipopolysaccharides (LPS) (**B**) by ELISA. Eachdot represents the result from a single animal. The horizontal line within each dataset denotes the average value. *p < 0.05; **p < 0.01; ****p < 0.0001 in a Tukey multiple comparison test which followed a one- way ANOVA.

To confirm compromised intestinal barrier integrity as indicated by changes in expression levels of genes encoding tight junction proteins (Fig. 3), we measured serum levels of the tight junction protein zonulin and gut bacterial lipopolysaccharides (LPS), since if intestinal integrity had been disrupted, we would observe elevated levels of both substances. Serum zonulin and LPS levels were measured using an ELISA, which has been successfully employed in clinical settings for both substances (Dutta et al. 2019; Zheng et al. 2021; Hoshiko et al. 2021a, b a, Hoshiko et al. 2021a, b b, Yuan et al. 2021; Niewiem et al., 2022). Analysis of zonulin revealed that exposure to the glyphosate, dicamba, and 2,4-D mixture resulted in a significant (*p* < 0.0001) 8-fold average increase in serum levels of this protein in females and a 2-fold but still significant (*p* < 0.05) average increase in males (Fig. 4A). The 4-fold difference in serum zonulin levels between male and female animals was also statistically significant (*p* < 0.0001; Supplementary Fig. 4). The greater disruption of intestinal barrier integrity in female animals exposed to the herbicide mixture was confirmed by the analysis of serum for bacterial LPS concentrations, which were significantly (*p* < 0.01) elevated from an average of 1.3ng/mL in the untreated control group to an average of 1.308ng/mL (Fig. 4B).

Exposure to the glyphosate NOAEL also resulted in a significant (*p* < 0.01) increase in serum zonulin levels in females of approximately 3-fold but with no increase in males (Fig. 4A). The difference in serum zonulin levels between male and female animals was also statistically significant (*p* < 0.01; Supplementary Fig. 4). No change in serum zonulin was detected in either female or male animals exposed to the glyphosate ADI (Fig. 4A). An increase in serum LPS concentrations to an average of 1.305ng/mL was observed in female rats exposed to the glyphosate NOAEL, but this did not reach statistical significance (Fig. 4B).

### Oxidative stress

**Fig. 5:**
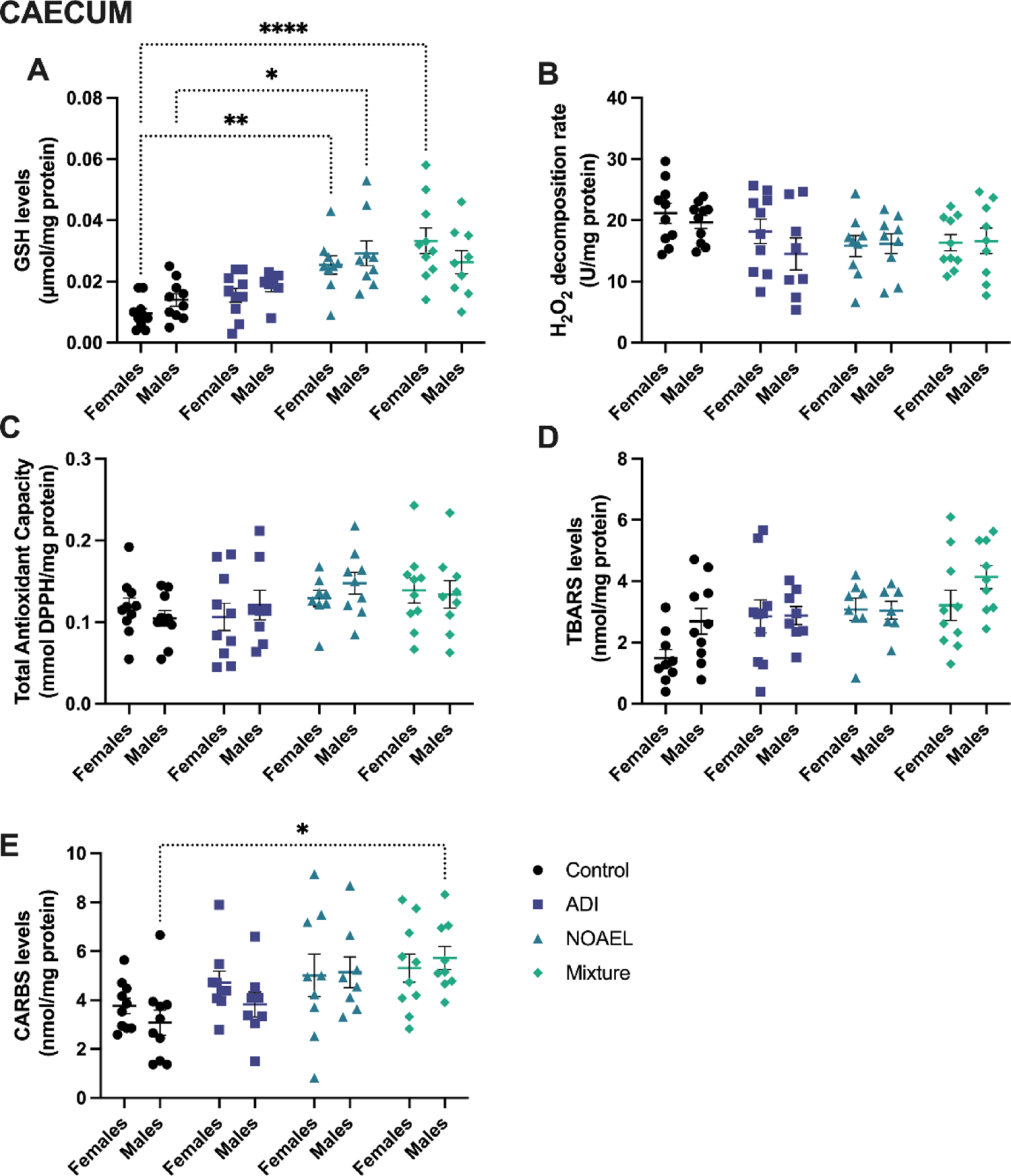
**Effect of glyphosate and glyphosate, 2,4-D, dicamba mixture on redox biomarkers in large intestine.** Large intestine from rats exposed to the glyphosate acceptable daily intake (ADI) and no observed adverse effect level (NOAEL), and ADI MIXTURE of glyphosate, dicamba, 2,4-D MIXTURE starting at GD6 until 13 weeks post-weaning was analysed for antioxidantdefense (GSH, Η_2_ Ο_2_ decomposition rate, TAC) and in oxidative damage (TBARS, CARBS). **A** : GSH concentration. **B** : Η 2 Ο 2 decomposition rate. **C** : TAC levels. **D** : TBARS levels. **E** : CARBS levels. Each dot represents the measurement from a single animal. *p < 0.05; **p < 0.01; ****p < 0.0001 in a Tukey multiple comparison test which followed a one- way ANOVA

Collectively, our measurements showed a more pronounced inflammation (Figs. 1 and 2) and compromised integrity (Figs. 3 and 4) in the large intestine compared to the ileum in response to exposure to the glyphosate NOAEL and the glyphosate, 2,4-D and dicamba mixture (Supplementary Tables 1 and 2). One mechanism through which such compromised gut structural and functional integrity could be brought about is induction of redox imbalance causing oxidative stress (Li et al. 2023a, Li et al. 2023b). In order to test this possibility, the levels of reduced glutathione (GSH), decomposition rate of Η_2_Ο_2_, total antioxidant capacity (TAC), thiobarbituric reactive substances (TBARS), and protein carbonyls (CARBS) were determined to see if there was an effect on antioxidant capacity and oxidative stress in the large intestine (Fig. 5), as we have previously observed in the liver of the same set of animals (Nechalioti et al. 2023). Reduced GSH levels were significantly (*p* < 0.01) increased in both female (average 166% increase) and male (108% average increase) animals exposed to the glyphosate NOAEL (Fig. 5A). A statistically significant (*p* < 0.0001) increase of 248% in reduced GSH was observed in female rats exposed to the glyphosate, 2,4-D and dicamba herbicide mixture; males showed a non-significant 87% increase when compared to untreated controls (Fig. 5A). The mixture of herbicides also induced a significant increase of 113% (*p* < 0.001) in GSH levels in females compared to the glyphosate ADI dose groups (Fig. 5A). CARBS levels were elevated by an average of 67% (*p* < 0.05) and 86% (*p* < 0.01) in groups of male animals treated with the glyphosate NOAEL and the glyphosate, 2,4-D and dicamba herbicide mixture in comparison to untreated controls (Fig. 5E). However, TBARS increased only in the herbicide mixture-treated groups, with similar effects in both female (115% increase; *p* < 0.05) and male (53% increase; *p* < 0.05) rats (Fig. 5D). This suggests that exposure to the mixture of herbicides caused oxidative stress, which resulted in peroxidation of intestinal membrane lipids. No difference between control and herbicide exposure groups was observed in the rate of decomposition of Η_2_Ο_2_ (Fig. 5B) and total antioxidant capacity (Fig. 5C).

### Sex-by-exposure interactions

We assessed if there were any sex-by-exposure interactions by undertaking ANOVA model analyses. This was deemed the most statistically valid manner to assess the influence of sex, because it uses the same statistical models that were used to calculate effects of herbicide exposure (Figs. 1, 2, 3, 4 and 5). Thus, all the comparisons are internally consistent and based on the same framework. The ANOVA analyses revealed distinct sex-specific effects in response to herbicide exposure (Supplementary Table 3), as demonstrated by significant interactions between sex and herbicide exposure in several parameters. For example, in the caecum, the interaction term accounted for 0.81% of the total variation in *Muc2* gene expression (*p* = 0.0113), while in the ileum, it explained 1.65% (*p* = 0.0082). Overall, interactions ranged from 0.25% to 11.54%, with the maximum observed for zonulin concentration in the serum.

Sex alone contributed substantially to the observed variation, with the sex factor explaining up to 4.49% of the variation in serum zonulin concentration (*p* = 0.0023) and 3.83% in *Il22* gene expression in the ileum (*p* = 0.0016). However, herbicide exposure effects dominated, explaining over 80% of the variation in most parameters, such as 92.83% of *Muc2* gene expression in the caecum (*p* < 0.0001).

### Bacterial and fungal gut microbiome composition and function

Another mechanism by which gut integrity can become compromised is through changes in composition of gut microbiota (Christovich and Luo 2022; Aleman et al. 2023; Di Tommaso et al. 2021). We investigated effects on bacterial and fungal communities, since we previously found that glyphosate affected fungi in the rat gut through decreased colonisation resistance (Mesnage et al. 2022). In the gut, fungi play a crucial role in maintaining gut homeostasis and overall health by contributing to nutrient metabolism, immune regulation, and the maintenance of a balanced microbial ecosystem (Huseyin et al. 2017).

**Fig. 6:**
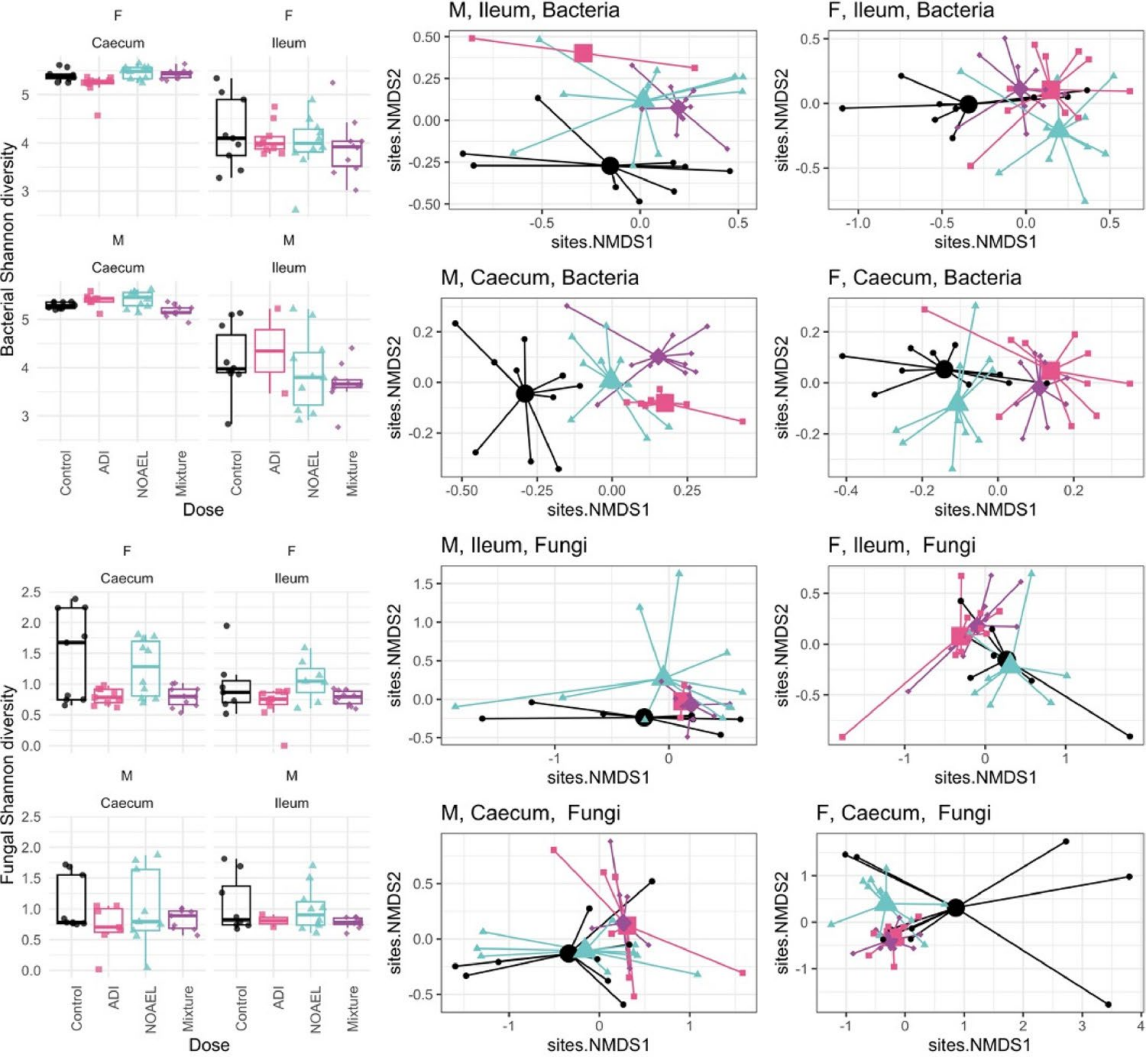
**Microbiome analysis of caecum and ileum content of rats exposed to glyphosate or glyphosate, 2,4-D, dicamba mixture.** Caecum and ileum from rats exposed from GD6 until 13-weeks post-weaning were analysed for gut microbiota composition. Alpha bacterial (top left panels) and fungal (bottom left panels) diversity were compared in the caecum or ileum in females(F) and males (M). Beta diversity for bacteria (top right panels) and fungi (bottom right panels) was measured using Bray-Curtis distances and represented.

We investigated bacterial diversity in caecum and ileum regions (Fig. 6). An ANOVA statistical test showed that the exposure to herbicides had no clear effect on bacterial diversity (*p* = 0.047), although a decrease in bacterial diversity was observed in groups of animals treated with the glyphosate, 2,4-D and dicamba mixture when compared to the control groups, though this did not reach statistical significance following a posthoc TukeyHSD test (*p* = 0.09) (Fig. 6, top left panels). There were no differences found between sexes and effects were comparable. However, bacterial diversity in the caecum was much higher than in the ileum (*p* = 0.0001) (Fig. 6, top left panels). Beta diversity calculated from Bray-Curtis distances revealed that the different groups separated well (*p* = 0.001), with the control group separating from the herbicide exposure groups (Fig. 6, top right-hand panels).

Changes in diversity were more evident in fungal populations. Alpha diversity of fungal populations in the gut microbiome of treated rats was lower for the group exposed to the glyphosate ADI (*p* = 0.0005) or the herbicide mixture (*p* = 0.0002) in comparison to untreated controls (Fig. 6, lower left hand panels). In the case of fungi, no role of gut compartment or sex was identified. Beta diversity was not evaluated for the fungal populations because the low number of species detected did not allow precise Bray Curtis distance calculations.

Bacteria at the phylum level were mostly Firmicutes, Bacteroidota and Proteobacteria, with a higher proportion of Firmicutes in the caecum than the ileum (Supplementary Table 4). The population of Bacteroidota was found to be higher in the ileum than the caecum. There were no changes observed in bacterial abundance, which was detectable and robust at the phylum level for bacteria (Supplementary Fig. 5). Most fungi in the gut microbiome were Ascomycota (> 99%) compared to the proportion of Basidiomycota, which was below 1% (Supplementary Fig. 6). No changes were detected in the proportions of these fungi between the different herbicide exposure groups (Supplementary Fig. 7).

**Fig. 7:**
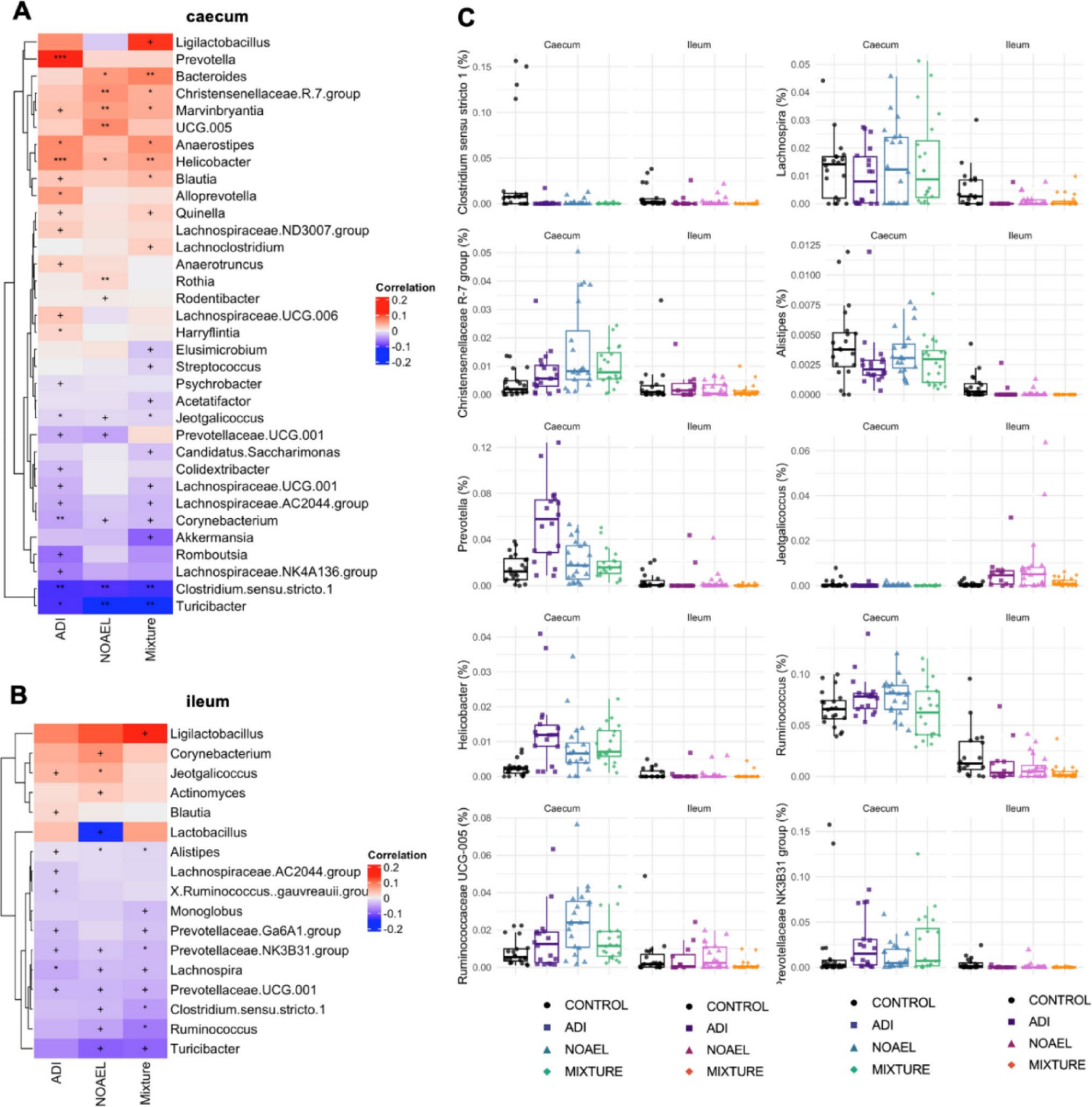
**Differentially abundant bacteria genera after exposure to glyphosate or glyphosate, 2,4-D, dicamba mixture. **Left hand panels: heatmaps showing whether the abundance of a bacteria genus is increased (red) or decreased (blue) in comparison to the control in caecum ( **A** ) or ileum ( **B** ). Right hand panels. ( **C** ): individual variations for the most differentially abundant genera are presented. Statistical significance was determined using linear-mixed models with the arcsin transformed abundance values using the animalID as a random effect. FDR (q-values) are used to determine statistical significance (+q<0.2, *q < 0.05; **q < 0.01; ****q < 0.0001). Each dot represents the measurement from a single animal

The changes in the gut microbiome of the rats exposed to glyphosate or the glyphosate, dicamba and 2,4-D mixture was more evident at the genus level (Fig. 7). Since there was no clear difference between sexes, the analysis was not stratified by sex, in order to improve statistical power. However, the analysis was done separately for caecum and ileum compartments, which presented differences in their microbiota composition (Fig. 6). A total of 9, 8 and 9 bacterial genera were altered by exposure to the glyphosate ADI, glyphosate NOAEL or herbicide mixture, respectively in the caecum (Fig. 7A). There were fewer changes in the ileum, with 1, 2 and 4 bacteria generally disturbed by the glyphosate ADI, NOAEL or herbicide mixture exposures, respectively (Fig. 7B). The exposure that produced the strongest changes in bacterial abundance was that to the herbicide mixture, with an increase in Bacteroides or Helicobacter, some strains within these genera being associated with proinflammatory responses. This was concomitant to a decrease in the levels of Lachnospira, Akkermansia, Ruminococcus and Alistipes, which includes known producers of short chain fatty acids (SCFAs) that are generally associated with a more balanced and anti-inflammatory gut environment. Overall, this shift in microbiota profile suggests the establishment of a pro-inflammatory environment.

To obtain insight into any metabolic functional consequences stemming from the alterations in microbiota composition in response to exposure with the herbicides (Figs. 6 and 7), analysis of the bacterial 16 S rRNA sequence dataset was undertaken with Picrust2 (Douglas et al. 2020). The results obtained showed that none of the MetaCyc pathways related to fermentation to butanoate (PWY-5022, PWY-5676, P162-PWY, P163-PWY, CENTFERM-PWY, PWY-5677) showed statistically significant differences in herbicide treatment groups compared to controls (Supplementary Table 5).

Nevertheless, changes in MetaCyc pathway abundance were found for 66, 22, and 2 pathways in the comparison between the control group and groups exposed to the glyphosate ADI, glyphosate NOAEL, and herbicide mixture, respectively, with adjusted p-values as low as 6.8 × 10^−5^ (Supplementary Table 6).

Exposure to glyphosate resulted in significant disruptions to several key metabolic pathways affecting energy metabolism. Notably, pathways involved in nucleotide metabolism were affected, including the superpathways of purine and pyrimidine deoxyribonucleoside degradation, and de novo biosynthesis pathways I & II for adenosine nucleotides. In addition, amino acid biosynthesis pathways, particularly those related to lysine, were significantly different. The tRNA charging pathway, essential for protein synthesis, was also disrupted. Carbohydrate metabolism pathways including sucrose degradation III (sucrose invertase), glycolysis I (from glucose 6-phosphate), glycolysis III (from glucose), and homolactic fermentation, showed notable differences between herbicide exposure and control groups. Furthermore, pathways crucial for cell wall and lipid metabolism were affected, such as peptidoglycan biosynthesis I (meso-diaminopimelate containing), peptidoglycan biosynthesis III (mycobacteria), CDP-diacylglycerol biosynthesis I & II, and gondoate biosynthesis (anaerobic).

### Histopathological analysis

Histological analysis was undertaken to see if the abnormalities of inflammation and compromised gut integrity predicted by biochemical measures (Figs. 1, 2, 3 and 4) could be seen at a tissue and cellular level. Results from the scoring system employed to classify pathologies from absent to intense are collated in Tables 1 and 2. Representative images of histological sections showing the main pathologies observed are shown in Supplementary Figs. 8–11.

**Table 1:** **Histological analysis of rat caecum following exposure to the EU glyphosate acceptable daily intake (ADI), glyphosate no observed adverse effect level (NOAEL) and ADI mixture (MIXT) of glyphosate, dicamba, 2,4-D**

Parameters	Experimental groups																
	Control				NOAEL				ADI					MIXT			
	Intensity		Specimens number		Intensity		Specimens number		Intensity			Specimens number		Intensity		Specimens number	
	F	M	F	M	F	M	F	M	F		M	F	M	F	M	F	M
Modified cell aspect	−	−	0/10	0/10	+	+	4/10*	5/10*	−/+		+	3/10	3/8*	−/+	++	2/10	6/10*
Chronic inflammation	−	−	0/10	0/10	−/+	−	2/10	10/10*	+		+ +	4/10*	5/8*	+	+	5/10*	4/10*
Vessel appearance: dilatation, hyperemia	−	−	0/10	0/10	+	+	5/10*	5/10*	+ +	+ +		5/10*	5/8*	+ + +	+	7/10*	3/10

Lesion severity was graded as (−) absent, (+) mild, (+ +) moderate, (+ + +) intense. F, female; M, male. *p-value < 0.05 compared to control groups by chi-square test

**Table 2:** **Histological analysis of rat ileum following exposure to the EU glyphosate acceptable daily intake (ADI), glyphosate no observed adverse effect level (NOAEL) and ADI mixture (MIXT) of glyphosate, dicamba, 2,4-D **

Parameters	Experimental groups																
	Control				NOAEL				ADI					MIXT			
	Intensity		Specimens number		Intensity		Specimens number		Intensity			Specimens number		Intensity		Specimens number	
	F	M	F	M	F	M	F	M	F		M	F	M	F	M	F	M
Modified cell aspect	−	−	0/10	0/10	-/+	-/+	4/10*	5/10*	+		+	4/10*	3/8*	-/+	-/+	2/10	3/10
Chronic inflammation	−	−	0/10	0/10	+ +	+ +	5/10*	5/10*	+ +		+	6/10*	3/8*	+ + +	+	7/10*	3/10
Vessel appearance: dilatation, hyperemia	−	−	0/10	0/10	+	+ +	4/10*	6/10*	+ +	+		5/10*	3/8*	+ +	+ +	5/10*	5/10*

Lesion severity was graded as (−) absent, (+) mild, (+ +) moderate, (+ + +) intense. F, female; M, male. *p-value < 0.05 compared to control groups by chi-square test

Normal architecture with no signs of apparent inflammation or injury was observed in the ileum and caecum from both females and males (Tables 1 and 2; Supplementary Figs. 8–11, panels A).

Histopathological results in the caecum are presented in Table 1 with key findings as follows. In the glyphosate NOAEL groups, mild severity was observed in 4/10 females and 5/10 males with mucosal crypt cell morphological changes (e.g. microvacuolar/vascular cytoplasm) (Supplementary Figs. 8B, 9B). Also, both female (5/10; Supplementary Fig. 8B) and male (5/10; Supplementary Fig. 9B) rats showed mild severity intramural dilatated blood vessels with congestion. The male glyphosate ADI group showed more obvious changes compared to females with mild modified cell aspect in 3/8 animals (microvacuolar cytoplasm), and moderate chronic inflammation in submucosa and dilated, hyperemic vessels in 5/8 cases (Supplementary Fig. 9C). In the female ADI group, mild chronic inflammation (4/10 cases) and moderate dilated hyperemic blood vessels, especially in submucosa (5/10 cases) was observed (Supplementary Fig. 8C). In the glyphosate, 2,4-D and dicamba mixture-exposed groups, the most pronounced changes in females were severe dilatated vessels with congestion with cases of margination of leucocytes to the blood vessel wall in 7/10 animals (Supplementary Fig. 8D). Mild focal inflammation was also present (4/10 cases). In males, the main changes were moderate modified cell aspect in mucosa with glands showing hypertrophic, hyperchromatic, slightly irregular nuclei and margination of chromatin in association with small hyperemic vessels (6/10 animals), and mild intramural chronic inflammation in 4/10 cases (Supplementary Fig. 9D). Statistical analysis of pathological scores in caecum showed a significant increase in the glyphosate NOAEL female group compared to controls regarding cell aspects and vessel appearance (*p* < 0.05; Table 1). In the case of the female glyphosate ADI and herbicide mixture groups, significant increases in pathologies related to inflammation and vessel appearance was also evident (*p* < 0.05; Table 1). In male animals, significant increases in pathological incidence were observed from exposure to the glyphosate NOAEL and ADI for all parameters, and for cell aspects and chronic inflammation in the herbicide mixture group compared to controls (*p* < 0.05; Table 1).

Histopathological outcomes in the ileum are shown in Table 2 with the following main results. In both female (Supplementary Fig. 10B) and male (Supplementary Fig. 11B) glyphosate NOAEL groups there were histological disruptions marked by a decreased height of the villi and moderate chronic inflammation in the villous core in 5/10 animals in each case. Red blood cells were also present. In the glyphosate ADI groups, both males (3/8 cases) and females (4/10 cases) showed a mild variation in modified cell aspect (cell size; presence of microvacuolar cytoplasm; Supplementary Fig. 10C, 11C). There was moderate vascular congestion and focal chronic inflammation in the villi of females in 6/10 animals (Supplementary Fig. 10C). In males, a mild severity in modified cell aspect (size of the muscularis propria) and vessel appearance (slight thickening of the walls and hyperemia) was observed in 3/8 cases (Supplementary Fig. 11C). Females showed a moderate (6/10 animals) and males a mild (3/8 cases) chronic inflammatory response with an infiltrate consisting mostly of lymphocytes as revealed by the presence of cells with dark blue round nuclei and inconspicuous cytoplasm (Supplementary Figs. 10C, 11C). The most pronounced pathologies in the glyphosate, 2,4-D and dicamba mixture groups were seen in females with severe chronic inflammation (7/10 animals) in the lamina propria, which was associated with moderate dilated vessels and congestion in 5/10 cases (Supplementary Fig. 10D). In males, mild focal chronic inflammation (3/10 cases) and moderate dilatated villi and frequent small hyperemic vessels (5/10 cases) was observed (Supplementary Fig. 11D). Statistical analysis of pathological scores in the ileum showed significantly increased values for cell aspects, chronic inflammation and altered vessel appearance in the female glyphosate NOAEL and ADI groups with a significant increase in chronic inflammation and altered vessel appearance also observed in the herbicide mixture group compared to controls (*p* < 0.05; Table 2). Similar statistically significant increases in pathologies were observed in male rats, with the small increase in altered vessel appearance in the herbicide mixture group also reaching significance (*p* < 0.05; Table 2).

## Discussion

This study comprises the most comprehensive investigation of the impact of glyphosate on gut structure and function, and the first to investigate potential toxicity of glyphosate in combination with 2,4-D and dicamba in a rat model system at regulatory relevant (EU ADI, NOAEL) doses deemed to be safe. Our results demonstrate that in contrast to glyphosate alone at the EU ADI dose, exposure of rats to a mixture of glyphosate, dicamba and 2,4-D, with each at their respective EU ADI, induced gut inflammation and permeability linked with a leaky gut condition. These effects were associated with an oxidative stress response and alterations in microbial community structure. Glyphosate alone at the EU NOAEL also gave rise to similar, though less prominent, outcomes in terms of gut inflammation and integrity, but which again were associated with an induced oxidative stress response. Histopathological analysis confirmed structural alterations and inflammation in the large intestine and ileum as predicted by the biochemical measures. Effects were more pronounced in female than male animals. Thus, our findings reveal significant alterations in gut microbial communities and associated gut functions, with implications for intestinal health.

A notable outcome of this study is the increased expression of *Il22*, *Lcn2* and *Tlr4*, which encode key inflammatory mediators, in the large intestine and ileum of male and female animals exposed to the herbicide mixture and the glyphosate NOAEL (Fig. 1), but which was more pronounced in female than male animals (Supplementary Fig. 2). Concomitant to the increase in expression of inflammatory marker genes was decreased expression of genes associated with intestinal permeability, namely *Muc2*, *Ocln*, *Zo-1*, *Cldn3* and *Cldn4* again detectable in the large intestine and ileum of male and female animals exposed to the herbicide mixture and the glyphosate NOAEL (Fig. 3). These alterations in gene expression patterns were greater in female rats and those exposed to the herbicide mixture suggesting that the combination of glyphosate, dicamba, and 2,4-D at a regulatory permitted (ADI) dose can induce a pro-inflammatory state in the intestinal track, raising concerns about the potential for chronic inflammation. A marked increase in calprotectin in the large intestine of female rats confirmed the induction of a gut inflammatory response following exposure to the herbicide mixture (Fig. 2). Gut inflammation following exposure to glyphosate has previously been reported in rats (Tang et al. 2020), mice (Panza et al. 2021; Lehman et al. 2023) and piglets (Qiu et al. 2020). We document for the first time, changes in gene expression associated with intestinal permeability, which raise the possibility of compromised intestinal integrity, allowing harmful substances to pass through the intestinal lining, potentially contributing to systemic inflammation and health complications (Jayashree et al. 2014).

Our finding of increased serum levels of both the intestinal tight junction protein zonulin and gut bacterial-derived LPS in rats exposed to the mixture of glyphosate, 2,4-D and dicamba (Fig. 4), which in the case of zonulin was significantly higher in female rats compared to males (Supplementary Fig. 4), provides consistent evidence of disrupted gut barrier function and supports the notion of increased systemic inflammation, reinforcing the significance of our findings. This observation may have long-term health consequences since elevated levels in blood of zonulin and LPS indicative of compromised intestinal barrier integrity has been linked with clinical conditions such as Graves’ disease (Zheng et al. 2021), childhood food allergies (Niewien et al., 2022) and metabolic dysfunction (Hoshiko et al. 2021a, b a and 2021 b). Furthermore, gut bacterial fragments in the systemic circulation are associated with the development of a variety of chronic conditions, including atherosclerosis (Violi et al., 2023), fatty liver disease (Kim and Ko 2018), type 2 diabetes (Jayashree et al. 2014; Yuan et al. 2021) and cancer (Li et al. 2023a, b).

Our measures of antioxidant capacity and oxidative stress markers in the large intestine (Fig. 5) highlight the impact of pesticide exposure on gut health. The increased levels of reduced GSH and CARBS indicate elevated oxidative stress, suggesting potential damage to cellular components and membrane lipids. The observed increase in TBARS levels in rats exposed to the herbicide mixture further signifies lipid peroxidation and oxidative damage. These observations are in accord with those detected in the liver of the same animals (Nechalioti et al. 2023). The induction of oxidative stress is one of the most characterised mechanisms by which glyphosate can induce toxic effects. This originates from disruption of mitochondrial function following glyphosate interactions with respiratory chain complexes (Chaufan et al. 2014; Bailey et al. 2018; Pereira et al. 2018). In addition, compelling evidence has linked exposure to 2,4-D with the induction of oxidative stress, as highlighted by the International Agency for Research on Cancer (Loomis et al. 2015). This herbicide is recognized as a potent oxidative stress-inducing agent, thus potentially occupying a central role in the observed toxic effects of the pesticide mixture in this study. This possibility is supported by our observations in an in vitro study using the ToxTracker cell culture assay system, where 2,4-D but not dicamba or glyphosate gave a clear oxidative stress response (Mesnage et al. 2021a). Furthermore, an epidemiological study has described a substantial and significant increase in molecular oxidative stress markers in the urine of farm workers within 24 h of application of a GBH (Chang et al. 2023) suggesting that observations in animal models as presented here can also occur in humans.

The investigation into bacterial and fungal microbiome composition provides insight into the impact of pesticide exposure on gut microbial communities (Tsiaoussis et al. 2019). The limited effects observed at the bacterial diversity level imply that overall bacterial richness and diversity were minimally influenced by exposure to the herbicides. However, changes at the genus level, particularly higher levels of Helicobacter and Alloprevotella in the herbicide mixture-exposed group, includes some strains known to contribute to proinflammatory states (Hansen eta l. 2011, Valour et al. 2014), suggesting that microbial composition was influenced, potentially altering the balance of commensal and pathogenic microorganisms. This was concomitant with a decrease in the levels of bacteria which are known SCFA producers, namely Lachnospira (Abdugheni et al. 2022), Eubacterium spps (Louis et al. 2010), Intestinomonas (Kläring et al. 2013) and Alistipes (Parker et al. 2020). A decrease in abundance of these bacterial species may lead to a reduction in SCFA levels, potentially contributing to increased inflammation as observed in this study. We were unable to confirm compromised SCFA production from the changes in bacterial species we observed employing the PICRUSt2 computational tool, which can predict functional changes associated with microbiome alterations (Douglas et al. 2020). This may be because PICRUSt2 is optimised for use with human microbiome datasets and is less suited for deriving toxicological insights in rats, whose microbiota are poorly characterized with respect to function. Nevertheless, the PICRUSt2 analysis predicted disruption of several key metabolic pathways from exposure to glyphosate, such as those involved in energy metabolism, nucleotide metabolism, purine and pyrimidine deoxyribonucleosides degradation, and de novo biosynthesis pathways I & II for adenosine nucleotides (Supplementary Tables 5 and 6). However, the toxicological relevance of these predicted metabolic changes remains uncertain as they need confirmation by direct experimentation such as untargeted metabolomics. We previously demonstrated in a rat model system the ability of glyphosate and glyphosate-based herbicides to alter the composition and function of the gut microbiome using metagenomics and metabolomics respectively, with metabolomics revealing induction of oxidative stress (Mesnage et al. 2021c). In a subsequent investigation we showed that glyphosate and glyphosate-based herbicide exposure in rats starting prenatally markedly altered the bacterial microbiota population, favouring growth of opportunistic fungi (Mesnage et al. 2022).

The two studies published to date reporting the effects of 2,4-D on the murine microbiome showed an enrichment in pro-inflammatory bacteria, such as Deferribacteres (Romualdo et al. 2023) and Spirochaetes (Tu et al. 2019). The levels of these two bacterial species were unchanged in our study, possibly due to differences in response between mice and rats. In contrast, to our knowledge there are no studies investigating the effects of dicamba in the gut microbiome. Although it is not possible to disentangle the contribution of the individual compounds resulting from exposure to the glyphosate, 2,4-D, dicamba mixture, our study nevertheless identifies for the first time possible markers for the effects of the environmentally relevant mixture of herbicides tested in this investigation on gut microbiota populations.

The findings that gut function and microbiota composition are altered corresponds to the aetiology of gut dysbiosis in humans. In a healthy gut, the equilibrium in redox status, via the controlled release of electron acceptors such as oxygen, regulates microbial fuel sources (Litvak et al. 2018). Oxidative stress during inflammation can fuel harmful bacteria, potentially impeding the growth of beneficial microbes, leading to dysbiosis. This in turn can cause fragility of the gut barrier, promoting leaky gut. This suggests that effects of the tested herbicides on gut microbiota could originate in their ability to alter redox reactions, as shown in numerous studies (Mesnage and Antoniou 2021).

A limitation of our study is that we only tested a mixture of glyphosate, 2,4-D and dicamba, which does not allow a discernment of whether the gut dysbiosis observed was due to the action of all three herbicides or a sub-group (glyphosate plus 2,4-D, glyphosate plus dicamba, dicamba plus 2,4-D). Also, this knowledge gap does not provide insight into mixture toxicology, that is whether additive or synergistic interactions are taking place between the herbicides tested. Furthermore, not including assessment of representative commercial herbicide formulations of glyphosate, dicamba and 2,4-D as used in agricultural and non-agricultural settings, also constitutes a limitation, as it does not allow assessment of the toxicological contribution of the co-formulants present in such products (Mesnage and Antoniou 2018; Mesnage et al. 2019). However, this lack of information does not detract from the health implications of our findings since our study was specifically designed to reflect regulatory agency practice and an escalating real-world scenario where humans are exposed to all three herbicides. Thus, from this perspective whether a combination of all three or just two of the herbicide active ingredients lead to gut ill-health is a secondary, albeit interesting, consideration. Another limitation of our investigation is that it focused on a single time point (13 weeks post-weaning with exposure having been initiated mid-gestation), and as result does not address potential long-term effects of these herbicides on gut health. Indeed, the animals at the end of the period of herbicide exposure did not show overt outward signs of ill health. However, the fact that we observed clear alterations in gut structure and function indicative of compromised intestinal integrity after only a subchronic exposure period adds to the significance of our findings, as it suggests that a longer exposure period could result in negative gut health impacts, with consequences for the function of other physiological systems. Nevertheless, chronic (1–2 year) exposure periods are needed to fully comprehend the trajectory and persistence of observed alterations in gut health (Docea et al. 2018).

Our findings may also have regulatory implications. First, given the recognised importance of gut structure and functional integrity in health and disease, our results add to the growing body of evidence of the importance of incorporating measurements such as those we present here as part of regulatory pesticide risk assessment. Second, our observations that exposure to the glyphosate, dicamba and 2,4-D mixture, with each herbicide at its EU ADI dose, caused pronounced alterations in gut structure and function build on previous work (Mesnage et al. 2021b, d). Together, these results show that ADI values based on risk assessment of an individual pesticide, as in current regulatory practice, can be incorrect – with public health implications.

## Conclusion

This investigation provides important insights into the potential hazards to health posed by glyphosate and its mixture with dicamba and 2,4-D on gut structure, microbiota composition and physiological function. The observed alterations in inflammatory markers, barrier integrity, oxidative stress, and microbiome composition suggest a multifaceted impact on gut structure and function, including induction of compromised intestinal barrier integrity. These outcomes were more pronounced in the large intestine and female animals suggesting differential gastrointestinal tract region and sex sensitivity. However, further research is needed to elucidate the mechanisms through which these pesticides exert their effects on the gut, as well as potential long-term health risks. Our results are especially relevant to the US, which is experiencing a continuing increase in application of 2,4-D and dicamba along with glyphosate and consequent increased human exposure (Daggy et al. 2024; Gillezeau et al. 2020) with currently unknown health implications. In summary, our observations demonstrate that continued research is warranted to better understand the long-term implications of exposure to pesticide mixtures to human and environmental health.

## Supplementary Information

Below is the link to the electronic supplementary material.

Supplementary Material 1
