# Supplementary material for: Impact of glyphosate and its mixture with 2,4-D and dicamba on gut biochemical function, intestinal barrier integrity and microbiome composition in adult rats with prenatal commencement of exposure
Source: Arch Toxicol. 2026 Apr 28;100(8):3699–718. doi: 10.1007/s00204-026-04409-9 (PMC13379462; doi:10.1007/s00204-026-04409-9)
Supplement: Supplementary file 1 — Supplementary Material 1 [file 204_2026_4409_MOESM1_ESM.docx]

**SUPPLEMENTARY MATERIAL**

| **Gene** | **Large Intestine** | | | **Ileum** | | |
| --- | --- | --- | --- | --- | --- | --- |
|  | **ADI** | **NOAEL** | **MIXTURE** | **ADI** | **NOAEL** | **MIXTURE** |
| ***Il22*** | 0.98 ± 0.56 | 1.13 ± 0.70 | 7.86 ± 2.23 | 1.30 ± 0.87 | 1.21 ± 0.88 | 4.69 ± 2.42 |
| ***Tlr4*** | 0.86 ± 0.59 | 2.80 ± 0.43 | 6.22 ± 1.22 | 1.34 ± 0.45 | 3.06 ± 0.70 | 8.14 ± 1.65 |
| ***Lcn2*** | 1.26 ± 0.19 | 6.61 ± 1.66 | 10.2 ± 3.11 | 1.17 ± 0.69 | 6.08 ± 1.90 | 6.73 ± 2.08 |
| ***Muc2*** | 1.01 ± 0.30 | 0.15 ± 0.04 | 0.06 ± 0.01 | 0.96 ± 0.20 | 0.32 ± 0.08 | 0.15 ± 0.05 |
| ***Ocln*** | 0.89 ± 0.30 | 0.18 ± 0.10 | 0.05 ± 0.01 | 1.13 ± 0.50 | 0.25 ± 0.09 | 0.14 ± 0.05 |
| ***Zo1*** | 1.13 ± 0.27 | 0.28 ± 0.07 | 0.06 ± 0.02 | 1.10 ± 0.28 | 0.33 ± 0.08 | 0.17 ± 0.07 |
| ***Cldn3*** | 1.02 ± 0.43 | 0.14 ± 0.03 | 0.04 ± 0.008 | 1.17 ± 0.53 | 0.35 ± 0.06 | 0.13 ± 0.03 |
| ***Cldn4*** | 0.83 ± 0.38 | 0.11 ± 0.04 | 0.05 ± 0.02 | 1.18 ± 0.48 | 0.08 ± 0.03 | 0.07 ± 0.02 |

**Supplementary Table 1.** Summary statistics of RT-qPCR assay outcomes in female rat ileum and large intestine for expression of markers of inflammation (*Il22*, *Tlr4*, *Lcn2*) and gut integrity (*Muc2*, *Ocln*, *Zo1*, *Cldn3*, *Cldn4*) in response to treatment with glyphosate at the EU acceptable daily intake (ADI) and no-observed adverse effect level (NOAEL) doses, and a glyphosate, 2,4-D and dicamba mixture (each at the EU ADI). Relative changes in untransformed gene expression values compared to control, untreated animals +/- standard deviation about the mean is shown.

| **Gene** | **Large Intestine** | | | **Ileum** | | |
| --- | --- | --- | --- | --- | --- | --- |
|  | **ADI** | **NOAEL** | **MIXTURE** | **ADI** | **NOAEL** | **MIXTURE** |
| ***Il22*** | 1.32 ± 0.39 | 0.90 ± 0.22 | 4.12 ± 1.07 | 1.32 ± 0.5 | 3.73 ± 1.10 | 4.59 ± 1.20 |
| ***Tlr4*** | 1.23 ± 0.31 | 2.48 ± 0.80 | 5.15 ± 1.48 | 1.08 ± 0.73 | 2.37 ± 0.57 | 4.54 ± 1.34 |
| ***Lcn2*** | 1.10 ± 0.21 | 4.26 ± 0.89 | 10.2 ± 3.11 | 0.97 ± 0.59 | 3.66 ± 0.77 | 5.01 ± 1.48 |
| ***Muc2*** | 1.43 ± 0.39 | 0.26 ± 0.09 | 0.09 ± 0.04 | 1.05 ± 0.18 | 0.49 ± 0.12 | 0.23 ± 0.05 |
| ***Ocln*** | 0.98 ± 0.34 | 0.22 ± 0.10 | 0.08 ± 0.02 | 1.03 ± 0.28 | 0.33 ± 0.05 | 0.24 ± 0.09 |
| ***Zo1*** | 1.06 ± 0.31 | 0.42 ± 0.14 | 0.13 ± 0.03 | 1.10 ± 0.18 | 0.43 ± 0.11 | 0.25 ± 0.10 |
| ***Cldn3*** | 0.90 ± 0.34 | 0.22 ± 0.08 | 0.06 ± 0.01 | 1.12 ± 0.34 | 0.43 ± 0.14 | 0.23 ± 0.05 |
| ***Cldn4*** | 1.18 ± 0.29 | 0.14 ± 0.04 | 0.06 ± 0.02 | 1.06 ± 0.23 | 0.21 ± 0.06 | 0.10 ± 0.03 |

**Supplementary Table 2.** Summary statistics of RT-qPCR assay outcomes in male rat ileum and large intestine for expression of markers of inflammation (*Il22*, *Tlr4*, *Lcn2*) and gut integrity (*Muc2*, *Ocln*, *Zo1*, *Cldn3*, *Cldn4*) in response to treatment with glyphosate at the EU ADI and NOAEL doses, and a glyphosate, 2,4-D and dicamba mixture (each at the EU ADI). Relative changes in untransformed gene expression values compared to control, untreated animals +/- standard deviation about the mean is shown.

| **Parameter** | **Compartment** | **Source of Variation** | **% of total variation** | **p-value** |
| --- | --- | --- | --- | --- |
| *Muc2* expression | Caecum | Interaction | 0.8066 | 0.0113 |
| *Muc2* expression | Caecum | Sex Factor | 2.075 | <0.0001 |
| *Muc2* expression | Caecum | Treatment Factor | 92.83 | <0.0001 |
| *Muc2* expression | ileum | Interaction | 1.654 | 0.0082 |
| *Muc2* expression | ileum | Sex Factor | 2.436 | <0.0001 |
| *Muc2* expression | ileum | Treatment Factor | 86.76 | <0.0001 |
| *Ocln* expression | Caecum | Interaction | 0.328 | 0.3516 |
| *Ocln* expression | Caecum | Sex Factor | 0.484 | 0.03 |
| *Ocln* expression | Caecum | Treatment Factor | 92.22 | <0.0001 |
| *Ocln* expression | ileum | Interaction | 2.045 | 0.0135 |
| *Ocln* expression | ileum | Sex Factor | 1.051 | 0.0178 |
| *Ocln* expression | ileum | Treatment Factor | 83.77 | <0.0001 |
| *Zo-1* expression | Caecum | Interaction | 2.665 | <0.0001 |
| *Zo-1* expression | Caecum | Sex Factor | 1.782 | <0.0001 |
| *Zo-1* expression | Caecum | Treatment Factor | 89.3 | <0.0001 |
| *Zo-1* expression | ileum | Interaction | 1.228 | 0.0939 |
| *Zo-1* expression | ileum | Sex Factor | 1.135 | 0.0156 |
| *Zo-1* expression | ileum | Treatment Factor | 84.36 | <0.0001 |
| *Cldn3* expression | Caecum | Interaction | 0.996 | 0.0009 |
| *Cldn3* expression | Caecum | Sex Factor | 0.5541 | 0.002 |
| *Cldn3* expression | Caecum | Treatment Factor | 94.15 | <0.0001 |
| *Cldn3* expression | ileum | Interaction | 1.948 | 0.0044 |
| *Cldn3* expression | ileum | Sex Factor | 1.11 | 0.0057 |
| *Cldn3* expression | ileum | Treatment Factor | 86.91 | <0.0001 |
| *Cldn4* expression | Caecum | Interaction | 0.2467 | 0.3784 |
| *Cldn4* expression | Caecum | Sex Factor | 0.6205 | 0.0065 |
| *Cldn4* expression | Caecum | Treatment Factor | 94.03 | <0.0001 |
| *Cldn4* expression | ileum | Interaction | 2.95 | <0.0001 |
| *Cldn4* expression | ileum | Sex Factor | 1.55 | <0.0001 |
| *Cldn4* expression | ileum | Treatment Factor | 89.76 | <0.0001 |
| Zonulin conc. | Serum | Interaction | 11.54 | <0.0001 |
| Zonulin conc. | Serum | Sex Factor | 4.494 | 0.0023 |
| Zonulin conc. | Serum | Treatment Factor | 52.27 | <0.0001 |
| Calprotectin conc. | Caecum | Interaction | 39.4 | <0.0001 |
| Calprotectin conc. | Caecum | Sex Factor | 18.73 | <0.0001 |
| Calprotectin conc. | Caecum | Treatment Factor | 38.45 | <0.0001 |
| *Il22* expression | Caecum | Interaction | 4.117 | 0.0002 |
| *Il22* expression | Caecum | Sex Factor | 0.8393 | 0.0356 |
| *Il22* expression | Caecum | Treatment Factor | 81.75 | <0.0001 |
| *Il22* expression | ileum | Interaction | 9.499 | <0.0001 |
| *Il22* expression | ileum | Sex Factor | 3.831 | 0.0016 |
| *Il22* expression | ileum | Treatment Factor | 61.29 | <0.0001 |
| *Lcn2* expression | Caecum | Interaction | 0.8444 | 0.5108 |
| *Lcn2* expression | Caecum | Sex Factor | 1.441 | 0.05 |
| *Lcn2* expression | Caecum | Treatment Factor | 72.39 | <0.0001 |
| *Lcn2* expression | ileum | Interaction | 1.032 | 0.2637 |
| *Lcn2* expression | ileum | Sex Factor | 1.834 | 0.009 |
| *Lcn2* expression | ileum | Treatment Factor | 79.59 | <0.0001 |
| *Tlr4* expression | Caecum | Interaction | 1.508 | 0.1706 |
| *Tlr4* expression | Caecum | Sex Factor | 0.002593 | 0.9252 |
| *Tlr4* expression | Caecum | Treatment Factor | 76.96 | <0.0001 |
| *Tlr4* expression | ileum | Interaction | 1.63 | 0.1463 |
| *Tlr4* expression | ileum | Sex Factor | 2.517 | 0.0046 |
| *Tlr4* expression | ileum | Treatment Factor | 75.57 | <0.0001 |
| GSH | Caecum | Interaction | 3.983 | 0.1971 |
| GSH | Caecum | Sex Factor | 0.2029 | 0.6223 |
| GSH | Caecum | Treatment Factor | 40.06 | <0.0001 |
| Η_2_Ο_2_ decomposition rate | Caecum | Interaction | 2.168 | 0.6408 |
| Η_2_Ο_2_ decomposition rate | Caecum | Sex Factor | 1.033 | 0.3725 |
| Η_2_Ο_2_ decomposition rate | Caecum | Treatment Factor | 11.26 | 0.0399 |
| TAC | Caecum | Interaction | 2.322 | 0.6331 |
| TAC | Caecum | Sex Factor | 0.1898 | 0.7084 |
| TAC | Caecum | Treatment Factor | 8.29 | 0.1147 |
| TBARS | Caecum | Interaction | 4.006 | 0.3415 |
| TBARS | Caecum | Sex Factor | 3.815 | 0.0764 |
| TBARS | Caecum | Treatment Factor | 18.71 | 0.0025 |
| CARBS | Caecum | Interaction | 2.249 | 0.5961 |
| CARBS | Caecum | Sex Factor | 0.4961 | 0.5196 |
| CARBS | Caecum | Treatment Factor | 20.62 | 0.0014 |

**Supplementary Table 3.** Analysis of interactions in the two-way ANOVA models applied to understand the effects of the treatment with glyphosate at the EU acceptable daily intake (ADI) and no-observed adverse effect level (NOAEL) doses, and a glyphosate, 2,4-D and dicamba mixture (each at the EU ADI). The source of variation lists the interaction between sex and treatment (Interaction) and the main effects of sex (Sex Factor) and treatment (Treatment Factor). Percentage (%) of Total Variation represents the proportion of total variation in gene expression explained by each source of variation. The p-value indicates the statistical significance of each factor.

| **Dose** | **Sex** | **Comp** | **Firmicutes** | **Bacteroidota** | **Actinobacteriota** | **Campylobacterota** | **Desulfobacterota** |
| --- | --- | --- | --- | --- | --- | --- | --- |
| ADI | F | Caecum | 53.0 ± 5.6 | 35.7 ± 6.8 | 0.6 ± 0.2 | 0.9 ± 1.0 | 2.3 ± 1.3 |
| ADI | F | Ileum | 80.6 ± 9.7 | 4.8 ± 5.6 | 7.8 ± 5.4 | 0.0 ± 0.0 | 0.1 ± 0.1 |
| ADI | M | Caecum | 56.9 ± 2.3 | 32.7 ± 2.2 | 0.6 ± 0.1 | 1.0 ± 0.3 | 3.3 ± 0.5 |
| ADI | M | Ileum | 81.1 ± 18.3 | 12.5 ± 17.2 | 2.7 ± 1.1 | 0.3 ± 0.4 | 1.7 ± 2.4 |
| Control | F | Caecum | 56.5 ± 6.1 | 33.0 ± 7.9 | 0.5 ± 0.2 | 0.2 ± 0.1 | 3.3 ± 1.3 |
| Control | F | Ileum | 80.1 ± 10.3 | 8.8 ± 10.3 | 6.5 ± 5.3 | 0.1 ± 0.1 | 0.9 ± 1.6 |
| Control | M | Caecum | 62.2 ± 3.6 | 29.2 ± 3.0 | 1.2 ± 1.0 | 0.1 ± 0.2 | 1.6 ± 0.6 |
| Control | M | Ileum | 85.1 ± 9.4 | 7.3 ± 8.4 | 4.0 ± 2.8 | 0.1 ± 0.1 | 0.5 ± 0.6 |
| Mixture | F | Caecum | 57.1 ± 4.5 | 32.9 ± 5.3 | 0.5 ± 0.2 | 0.5 ± 0.4 | 2.4 ± 0.6 |
| Mixture | F | Ileum | 89.4 ± 4.6 | 2.1 ± 2.3 | 4.4 ± 3.2 | 0.1 ± 0.1 | 0.4 ± 0.8 |
| Mixture | M | Caecum | 60.7 ± 4.8 | 32.9 ± 4.7 | 0.4 ± 0.1 | 0.8 ± 0.5 | 1.9 ± 0.9 |
| Mixture | M | Ileum | 91.0 ± 4.6 | 0.4 ± 0.4 | 6.5 ± 3.6 | 0.0 ± 0.0 | 0.1 ± 0.2 |
| NOAEL | F | Caecum | 53.6 ± 6.0 | 33.3 ± 3.7 | 0.8 ± 0.4 | 0.6 ± 0.8 | 5.4 ± 3.5 |
| NOAEL | F | Ileum | 75.8 ± 12.6 | 1.7 ± 1.9 | 14.4 ± 10.5 | 0.0 ± 0.0 | 0.3 ± 0.6 |
| NOAEL | M | Caecum | 58.7 ± 2.3 | 33.8 ± 2.8 | 0.6 ± 0.1 | 0.5 ± 0.3 | 2.3 ± 1.1 |
| NOAEL | M | Ileum | 84.6 ± 8.9 | 5.1 ± 7.4 | 7.0 ± 5.2 | 0.1 ± 0.2 | 0.3 ± 0.4 |
| **Dose** | **Sex** | **Comp** | **Verrucomicrobiota** | **Spirochaetota** | **Proteobacteria** | **Patescibacteria** | **Elusimicrobiota** |
| ADI | F | Caecum | 2.3 ± 3.3 | 1.0 ± 0.6 | 0.3 ± 0.3 | 0.3 ± 0.1 | 0.4 ± 0.3 |
| ADI | F | Ileum | 1.1 ± 1.5 | 0.0 ± 0.0 | 5.1 ± 8.6 | 0.2 ± 0.4 | 0.0 ± 0.0 |
| ADI | M | Caecum | 0.0 ± 0.0 | 3.0 ± 0.8 | 0.1 ± 0.3 | 0.4 ± 0.1 | 0.3 ± 0.2 |
| ADI | M | Ileum | 0.0 ± 0.0 | 0.2 ± 0.2 | 1.2 ± 1.1 | 0.2 ± 0.2 | 0.0 ± 0.0 |
| Control | F | Caecum | 2.6 ± 4.0 | 1.5 ± 1.0 | 0.2 ± 0.2 | 0.5 ± 0.2 | 0.2 ± 0.1 |
| Control | F | Ileum | 0.4 ± 1.1 | 0.0 ± 0.0 | 2.5 ± 2.1 | 0.5 ± 0.7 | 0.0 ± 0.0 |
| Control | M | Caecum | 0.4 ± 0.4 | 1.9 ± 1.8 | 0.7 ± 0.7 | 0.4 ± 0.3 | 0.6 ± 0.5 |
| Control | M | Ileum | 0.2 ± 0.3 | 0.4 ± 0.6 | 2.0 ± 1.9 | 0.1 ± 0.1 | 0.1 ± 0.1 |
| Mixture | F | Caecum | 0.2 ± 0.6 | 1.7 ± 0.9 | 0.2 ± 0.2 | 0.5 ± 0.2 | 0.3 ± 0.4 |
| Mixture | F | Ileum | 0.1 ± 0.3 | 0.0 ± 0.1 | 3.1 ± 3.5 | 0.3 ± 0.2 | 0.0 ± 0.0 |
| Mixture | M | Caecum | 0.0 ± 0.0 | 1.2 ± 1.3 | 0.2 ± 0.1 | 0.1 ± 0.1 | 0.1 ± 0.1 |
| Mixture | M | Ileum | 0.0 ± 0.0 | 0.0 ± 0.0 | 2.0 ± 2.0 | 0.0 ± 0.0 | 0.0 ± 0.0 |
| NOAEL | F | Caecum | 1.8 ± 2.4 | 1.0 ± 1.1 | 0.4 ± 0.4 | 0.6 ± 0.4 | 0.5 ± 0.5 |
| NOAEL | F | Ileum | 0.1 ± 0.2 | 0.0 ± 0.0 | 7.2 ± 7.9 | 0.2 ± 0.4 | 0.0 ± 0.0 |
| NOAEL | M | Caecum | 0.0 ± 0.0 | 1.2 ± 0.7 | 0.3 ± 0.4 | 0.3 ± 0.3 | 0.4 ± 0.3 |
| NOAEL | M | Ileum | 0.0 ± 0.0 | 0.0 ± 0.0 | 2.6 ± 2.4 | 0.1 ± 0.1 | 0.0 ± 0.0 |

**Supplementary Table 4.** Gut microbiota composition at the phylum level in response to treatment with glyphosate at the EU acceptable daily intake (ADI) and no-observed adverse effect level (NOAEL) doses, and a glyphosate, 2,4-D and dicamba mixture (each at the EU ADI). Microbiota composition was determined by 16S V3-V4 region sequencing. Data represents relative abundance of the different 16S amplicon sequence variants having their abundance agglomerated at the phylum level.

| **description** | **group** | **p_adjust** |
| --- | --- | --- |
| 4-aminobutanoate degradation V | ADI | 0.305 |
| 4-aminobutanoate degradation V | NOAEL | 0.748 |
| 4-aminobutanoate degradation V | Mixture | 0.087 |
| acetyl-CoA fermentation to butanoate II | ADI | 0.933 |
| acetyl-CoA fermentation to butanoate II | NOAEL | 0.995 |
| acetyl-CoA fermentation to butanoate II | Mixture | 0.886 |
| L-glutamate degradation V (via hydroxyglutarate) | ADI | 0.737 |
| L-glutamate degradation V (via hydroxyglutarate) | NOAEL | 0.954 |
| L-glutamate degradation V (via hydroxyglutarate) | Mixture | 0.891 |
| L-lysine fermentation to acetate and butanoate | ADI | 0.899 |
| L-lysine fermentation to acetate and butanoate | NOAEL | 0.978 |
| L-lysine fermentation to acetate and butanoate | Mixture | 0.822 |
| pyruvate fermentation to butanoate | ADI | 0.185 |
| pyruvate fermentation to butanoate | NOAEL | 0.608 |
| pyruvate fermentation to butanoate | Mixture | 0.786 |
| succinate fermentation to butanoate | ADI | 0.857 |
| succinate fermentation to butanoate | NOAEL | 0.912 |
| succinate fermentation to butanoate | Mixture | 0.995 |

**Supplementary Table 5.** Gut microbiota functional analysis in response to treatment with glyphosate at the EU acceptable daily intake (ADI) and no-observed adverse effect level (NOAEL) doses, and a glyphosate, 2,4-D and dicamba mixture (each at the EU ADI) fails to show any effects on the pathways linked to the production of short-chain fatty acids in the gut microbiota of the rats. Only statistically significance changes are displayed in this table. Functional analysis was inferred using Picrust2. Statistical significance was determined using the standard statistical analysis in Picrust2 using the DeSeq2 statistical method. The p-values were adjusted using the Benjamini–Hochberg procedure.

| **description** | **group** | **p_adjust** |
| --- | --- | --- |
| superpathway of N-acetylglucosamine, N-acetylmannosamine and N-acetylneuraminate degradation | ADI | 6.89E-05 |
| sucrose degradation III (sucrose invertase) | ADI | 6.89E-05 |
| L-lysine biosynthesis VI | ADI | 9.85E-05 |
| superpathway of pyrimidine nucleobases salvage | ADI | 9.85E-05 |
| glycolysis III (from glucose) | ADI | 0.0002 |
| dTDP-L-rhamnose biosynthesis I | ADI | 0.0002 |
| UMP biosynthesis | ADI | 0.0002 |
| galactose degradation I (Leloir pathway) | ADI | 0.0002 |
| adenosine ribonucleotides de novo biosynthesis | ADI | 0.0002 |
| coenzyme A biosynthesis I | ADI | 0.0002 |
| L-lysine biosynthesis III | ADI | 0.0002 |
| superpathway of adenosine nucleotides de novo biosynthesis II | ADI | 0.0002 |
| superpathway of adenosine nucleotides de novo biosynthesis I | ADI | 0.0003 |
| UDP-N-acetylmuramoyl-pentapeptide biosynthesis II (lysine-containing) | ADI | 0.0003 |
| peptidoglycan biosynthesis I (meso-diaminopimelate containing) | ADI | 0.0003 |
| peptidoglycan biosynthesis III (mycobacteria) | ADI | 0.0003 |
| UDP-N-acetylmuramoyl-pentapeptide biosynthesis I (meso-diaminopimelate containing) | ADI | 0.0003 |
| gluconeogenesis I | ADI | 0.0004 |
| O-antigen building blocks biosynthesis (E. coli) | ADI | 0.0004 |
| CDP-diacylglycerol biosynthesis I | ADI | 0.0004 |
| adenosine deoxyribonucleotides de novo biosynthesis II | ADI | 0.0004 |
| guanosine deoxyribonucleotides de novo biosynthesis II | ADI | 0.0004 |
| gondoate biosynthesis (anaerobic) | ADI | 0.0004 |
| CDP-diacylglycerol biosynthesis II | ADI | 0.0004 |
| tRNA charging | ADI | 0.0004 |
| pyruvate fermentation to acetate and lactate II | ADI | 0.0007 |
| guanosine ribonucleotides de novo biosynthesis | ADI | 0.0007 |
| superpathway of phospholipid biosynthesis I (bacteria) | ADI | 0.0009 |
| S-adenosyl-L-methionine cycle I | ADI | 0.0013 |
| superpathway of N-acetylneuraminate degradation | ADI | 0.0015 |
| phosphatidylglycerol biosynthesis I (plastidic) | ADI | 0.0016 |
| phosphatidylglycerol biosynthesis II (non-plastidic) | ADI | 0.0016 |
| inosine-5'-phosphate biosynthesis I | ADI | 0.0018 |
| cis-vaccenate biosynthesis | ADI | 0.0041 |
| glycolysis I (from glucose 6-phosphate) | ADI | 0.0053 |
| Bifidobacterium shunt | ADI | 0.0053 |
| fatty acid elongation -- saturated | ADI | 0.0053 |
| hexitol fermentation to lactate, formate, ethanol and acetate | ADI | 0.0053 |
| L-methionine biosynthesis III | ADI | 0.0054 |
| peptidoglycan maturation (meso-diaminopimelate containing) | ADI | 0.0089 |
| peptidoglycan biosynthesis IV (Enterococcus faecium) | ADI | 0.0096 |
| adenine and adenosine salvage III | ADI | 0.0096 |
| superpathway of pyrimidine ribonucleotides de novo biosynthesis | ADI | 0.0096 |
| teichoic acid (poly-glycerol) biosynthesis | ADI | 0.0096 |
| UDP-N-acetyl-D-glucosamine biosynthesis I | ADI | 0.0096 |
| TCA cycle VIII (helicobacter) | ADI | 0.0131 |
| superpathway of pyrimidine deoxyribonucleosides degradation | ADI | 0.0138 |
| L-lysine biosynthesis I | ADI | 0.0153 |
| polyisoprenoid biosynthesis (E. coli) | ADI | 0.0153 |
| pyrimidine deoxyribonucleotides de novo biosynthesis II | ADI | 0.0168 |
| heterolactic fermentation | ADI | 0.0169 |
| acetylene degradation | ADI | 0.0235 |
| superpathway of pyrimidine deoxyribonucleotides de novo biosynthesis (E. coli) | ADI | 0.0243 |
| homolactic fermentation | ADI | 0.0245 |
| purine ribonucleosides degradation | ADI | 0.0265 |
| aerobic respiration I (cytochrome c) | ADI | 0.0281 |
| superpathway of L-threonine biosynthesis | ADI | 0.0290 |
| superpathway of purine nucleotides de novo biosynthesis II | ADI | 0.0309 |
| L-methionine biosynthesis I | ADI | 0.0309 |
| superpathway of purine deoxyribonucleosides degradation | ADI | 0.0310 |
| glycolysis II (from fructose 6-phosphate) | ADI | 0.0311 |
| urate biosynthesis/inosine 5'-phosphate degradation | ADI | 0.0330 |
| superpathway of guanosine nucleotides de novo biosynthesis II | ADI | 0.0341 |
| mixed acid fermentation | ADI | 0.0494 |
| superpathway of purine nucleotides de novo biosynthesis I | ADI | 0.0494 |
| pentose phosphate pathway (non-oxidative branch) | ADI | 0.0499 |
| gluconeogenesis I | Mixture | 0.0380 |
| guanosine nucleotides degradation III | Mixture | 0.0472 |
| purine ribonucleosides degradation | NOAEL | 0.0037 |
| adenine and adenosine salvage III | NOAEL | 0.0090 |
| L-lysine biosynthesis VI | NOAEL | 0.0109 |
| superpathway of N-acetylglucosamine, N-acetylmannosamine and N-acetylneuraminate degradation | NOAEL | 0.0138 |
| superpathway of pyrimidine deoxyribonucleosides degradation | NOAEL | 0.0153 |
| sucrose degradation III (sucrose invertase) | NOAEL | 0.0211 |
| superpathway of purine deoxyribonucleosides degradation | NOAEL | 0.0211 |
| gondoate biosynthesis (anaerobic) | NOAEL | 0.0235 |
| glycolysis I (from glucose 6-phosphate) | NOAEL | 0.0265 |
| L-lysine biosynthesis III | NOAEL | 0.0290 |
| tRNA charging | NOAEL | 0.0310 |
| homolactic fermentation | NOAEL | 0.0380 |
| adenosine ribonucleotides de novo biosynthesis | NOAEL | 0.0410 |
| glycolysis III (from glucose) | NOAEL | 0.0437 |
| UDP-N-acetylmuramoyl-pentapeptide biosynthesis II (lysine-containing) | NOAEL | 0.0448 |
| UMP biosynthesis | NOAEL | 0.0459 |
| CDP-diacylglycerol biosynthesis I | NOAEL | 0.0494 |
| superpathway of adenosine nucleotides de novo biosynthesis II | NOAEL | 0.0494 |
| peptidoglycan biosynthesis III (mycobacteria) | NOAEL | 0.0494 |
| superpathway of adenosine nucleotides de novo biosynthesis I | NOAEL | 0.0494 |
| CDP-diacylglycerol biosynthesis II | NOAEL | 0.0494 |
| peptidoglycan biosynthesis I (meso-diaminopimelate containing) | NOAEL | 0.0499 |

**Supplementary Table 6.** Gut microbiota functional analysis in response to treatment with glyphosate at the EU acceptable daily intake (ADI) and no-observed adverse effect level (NOAEL) doses, and a glyphosate, 2,4-D and dicamba mixture (each at the EU ADI). Only statistically significance changes are displayed in this table. Functional analysis was inferred using Picrust2. Statistical significance was determined using the standard statistical analysis in Picrust2 using the DeSeq2 statistical method. The p-values were adjusted using the Benjamini–Hochberg procedure.

|  |  |  | ADI | | Mixture | |
| --- | --- | --- | --- | --- | --- | --- |
|  |  |  | M | F | M | F |
| Gut inflammation | *Il22* expression | Ileum | - | - | ↑ | ↑ |
|  |  | Caecum | - | - | ↑ | ↑ |
|  | *Lcn2* expression | Ileum | - | - | ↑ | ↑ |
|  |  | Caecum | - | - | ↑ | ↑ |
|  | *Tlr4* expression | Ileum | - | - | ↑ | ↑ |
|  |  | Caecum | - | - | ↑ | ↑ |
|  | Calprotectin conc. | Caecum | - | - | - | ↑ |
| Intestinal permeability | *Muc2* gene expression | Ileum | - | - | ↓ | ↓ |
|  |  | Caecum | - | - | ↓ | ↓ |
|  | *Ocln* gene expression | Ileum | - | - | ↓ | ↓ |
|  |  | Caecum | - | - | ↓ | ↓ |
|  | *Zo-1* expression | Ileum | - | - | ↓ | ↓ |
|  |  | Caecum | - | - | ↓ | ↓ |
|  | *Cldn3* expression | Ileum | - | - | ↓ | ↓ |
|  |  | Caecum | - | - | ↓ | ↓ |
|  | *Cldn4* expression | Ileum | - | - | ↓ | ↓ |
|  |  | Caecum | - | - | ↓ | ↓ |
|  | Zonulin conc. | Serum | - | - | ↑ | ↑ |
|  | LPS conc. | Serum | nd | - | nd | ↑ |
| Oxidative stress | GSH | Caecum | - | - | - | ↑ |
|  | Η_2_Ο_2_ decomposition rate | Caecum | - | - | - | - |
|  | TAC | Caecum | - | - | - | - |
|  | TBARS | Caecum | - | - | - | - |
|  | CARBS | Caecum | - | - | - | ↑ |

**Supplementary Table 7.** Summary of the main effects on gut inflammation, intestinal permeability of the glyphosate, 2,4-D and dicamba mixture (each at the EU ADI) in comparison to the glyphosate ADI dose alone. Hyphens, unchanged; ↑, statistically significant increase; ↓, statistically significant decrease; nd, not determined.


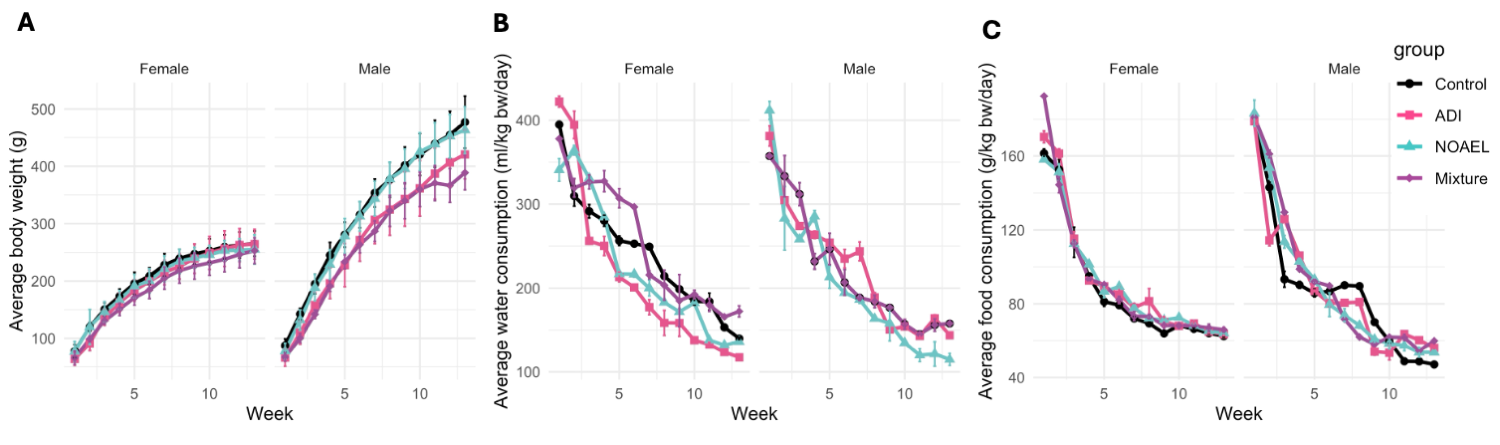


**Supplementary Fig. 1.** Changes in body weight (**A**), water consumption (**B**) and food consumption (**C**) after exposure to glyphosate at the EU acceptable daily intake (ADI) and no-observed adverse effect level (NOAEL), and a combination of glyphosate, 2,4-D and dicamba (Mixture) with each at the EU ADI. Data shown represents the changes in average values ± SD during the 13-week duration of the experiment for the 4 experimental groups with readings taken at weekly intervals. Statistical analysis was undertaken using linear mixed-effects models (lmerTest), which account for repeated measurements within the same animal, as follows. For each sex we fitted the model: outcome ~ week × group + (1/Animal), with week (time) and group as fixed effects and animal as a random intercept. This approach tests the main effects of time and group as well as the time × group interaction and addresses the same question as a two-way repeated-measures ANOVA, while appropriately modelling within-subject dependence and accommodating missing values.


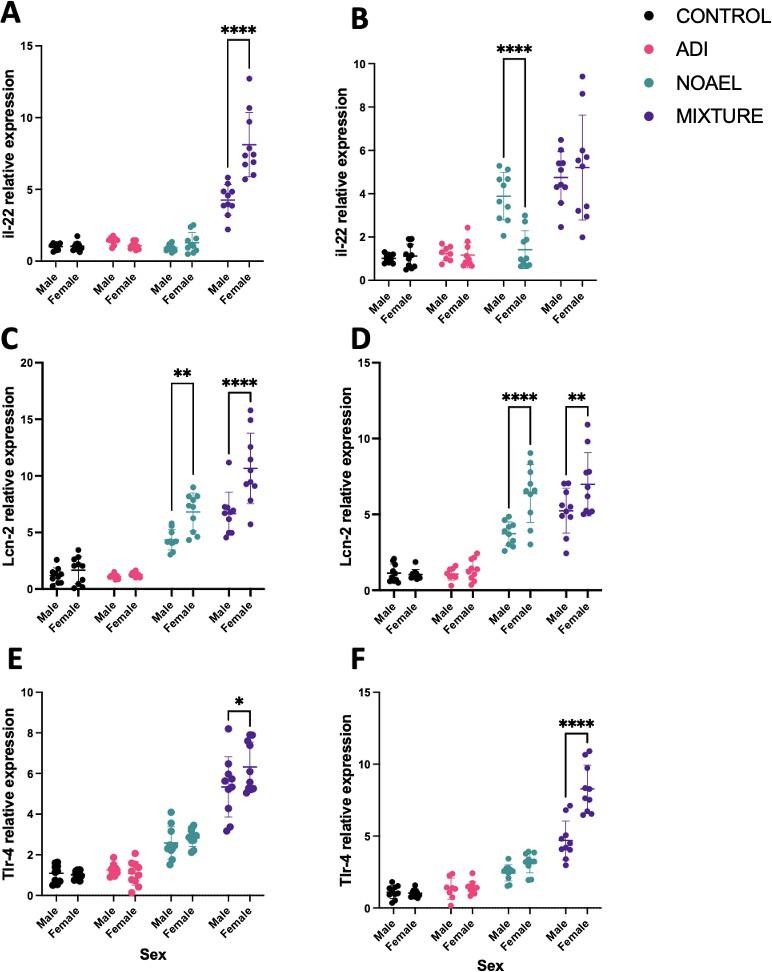


**Supplementary Figure 2.** Sex-specific effects of glyphosate or its mixture with dicamba and 2,4-D at regulatory permissible intake on genes associated with intestinal inflammation. A: *Il22* large intestine, B: *Il22* ileum, C: *Lcn2* large intestine, D: *Lcn2* ileum, E: *Tlr4* large intestine F: *Tlr4* ileum. Mixture groups of females had significantly increased expression compared to males of *Il22* in the large intestine (A; purple), Lcn2 in both the large intestine and ileum (C, D; purple) and *Tlr4* in both the large intestine and ileum (E, F; purple). Expression of *Lcn2* in the large intestine and ileum was increased in females compared to males exposed to glyphosate at the highest dose (C, D; green). The assay was performed in duplicate, and averages of the duplicates were plotted for each individual. Each dot represents the result from a single animal. The longer horizontal line within each set of data points denotes the average value for the group. *p <0.05; **p <0.01; ****p<0.0001 in a Šídák multiple comparison test which followed a one-way ANOVA.


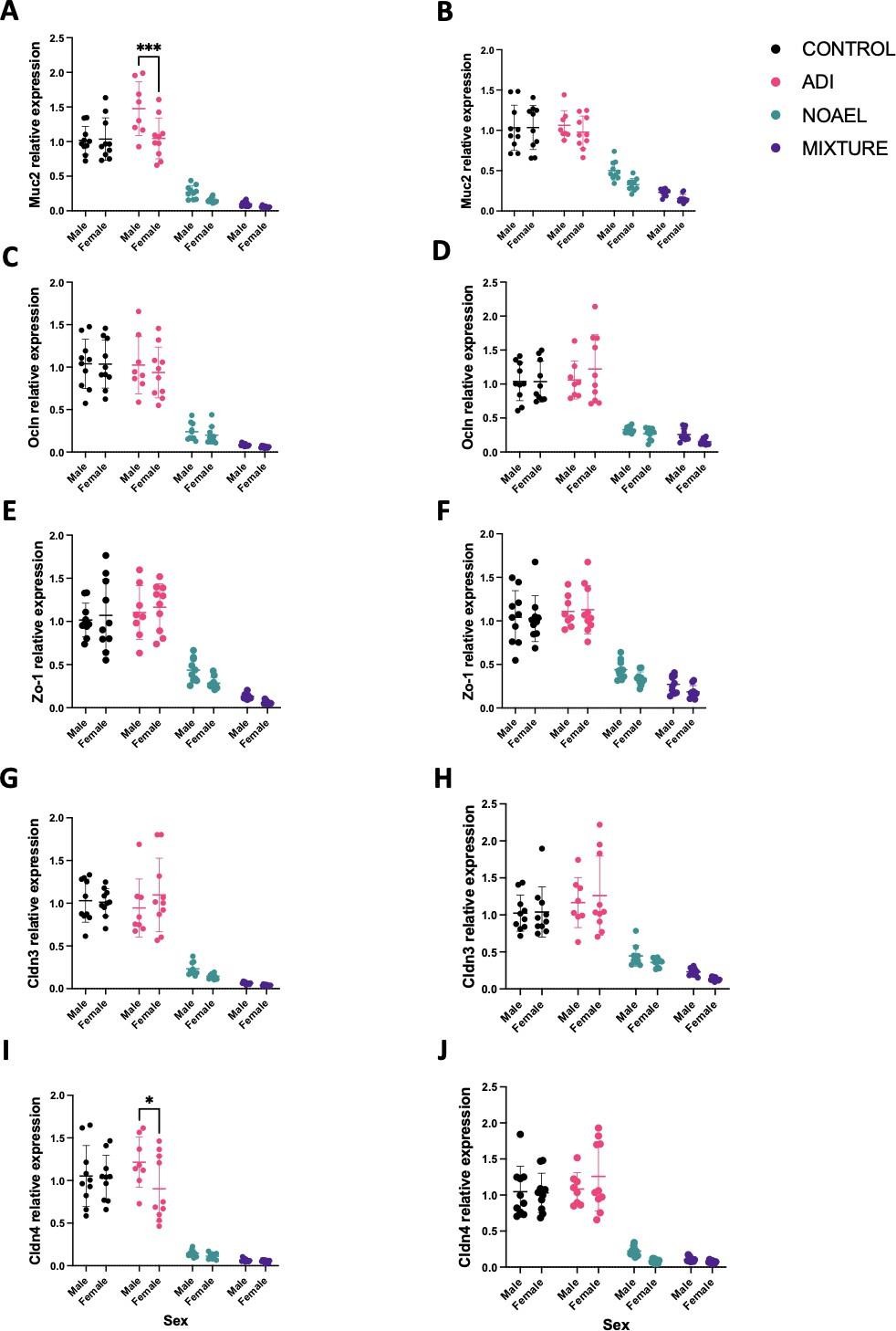


**Supplementary Figure 3.** Sex-specific effects of exposure to glyphosate at the EU ADI and NOAEL doses or its mixture with dicamba and 2,4-D (each at their EU ADI doses) on expression of genes associated with intestinal integrity. A: Muc2 large intestine, B: Muc2 ileum, C: Ocln large intestine, D: Ocln ileum, E: Zo-1 large intestine, F: Zo-1 ileum female, G: Cldn3 large intestine, H: Cldn3 ileum, I: Cldn4 large intestine, J: Cldn4 ileum. Between the ADI groups, large intestine expression of Muc2 (A; pink), and Cldn4 (I; pink) was significantly decreased in females compared to males. The assay was performed in duplicate, and averages of the duplicates were plotted for each rat. Each dot represents the result from a single animal. The longer horizontal line within each set of data points denotes the average value for the group. *p <0.05; **p <0.01; ***p <0.001; ****p<0.0001 in a Šídák multiple comparison test which followed a one-way ANOVA.


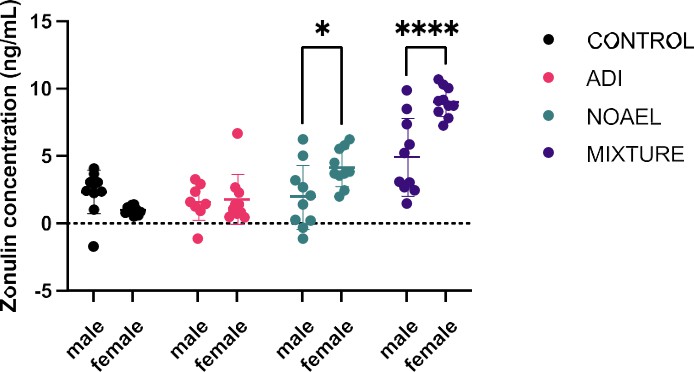


**Supplementary Figure 4.** Comparison of serum zonulin as an indicator of gut integrity status after exposure to glyphosate, 2,4-D and dicamba. Zonulin concentration was significantly increased in females exposed to glyphosate at its NOAEL dose (green) and females exposed to the mixture of glyphosate, dicamba and 2,4-D (purple) compared to males. The assay was performed in duplicate, and averages of the duplicates were plotted for each rat. The longer horizontal line within each set of data points denotes the average value for the group. *p <0.05; **p <0.01; ****p <0.0001 in a Šídák multiple comparison test which followed a one- way ANOVA.


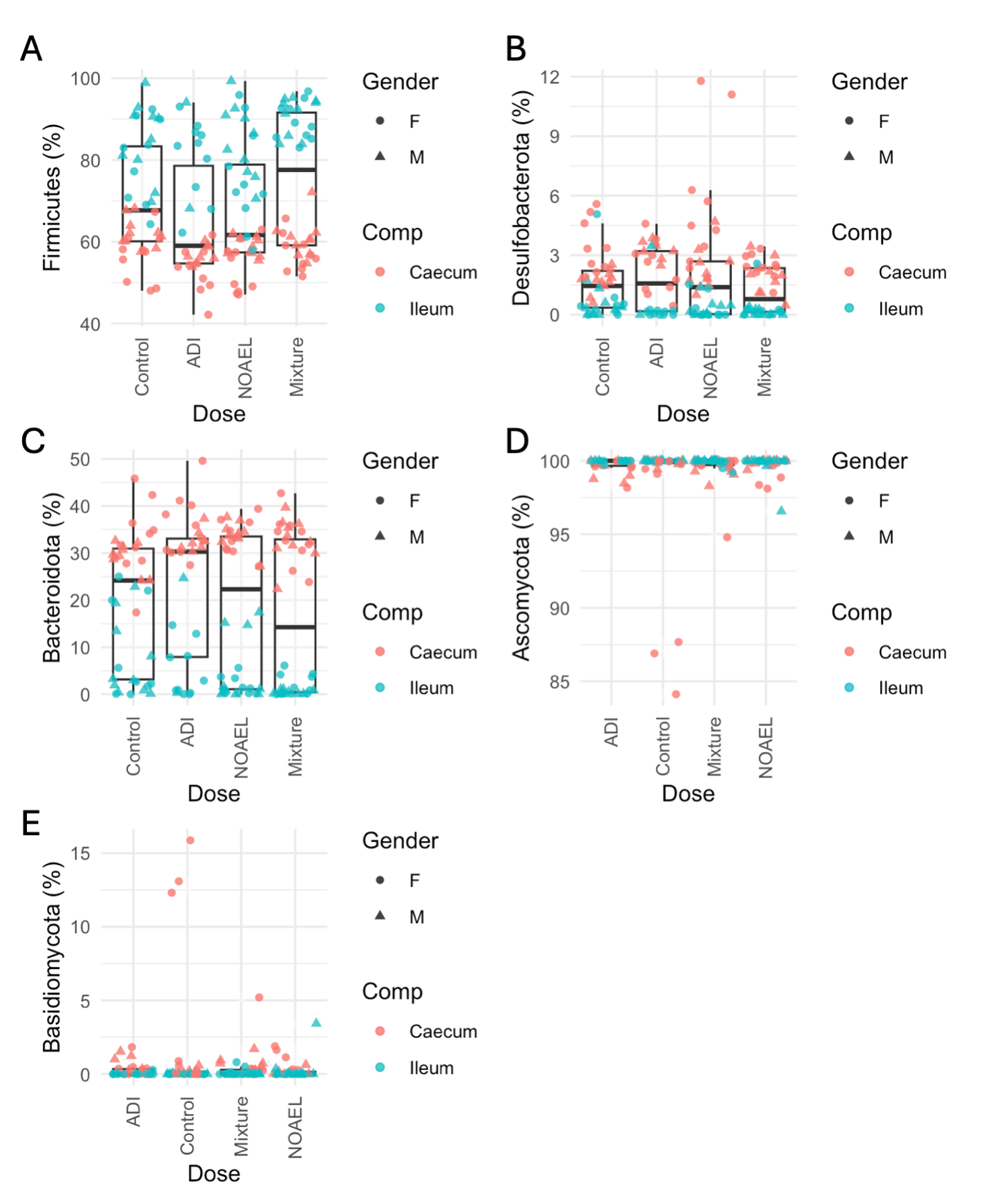


**Supplementary Figure 5.** Variation in gut bacterial phylum abundance in response to exposure with glyphosate at the EU acceptable daily intake (ADI) and no-observed adverse effect level (NOAEL) doses, and a combination of glyphosate, 2,4-D and dicamba (Mixture) with each at the EU ADI. Microbiota composition was determined by ITS2 region sequencing for fungi. Data represents relative abundance of the different 16S amplicon sequence variants having their abundance agglomerated at the phylum level. (**A**) Firmicutes, (**B**) Desulfobacterota, (**C**) Bacteroidota, (**D**) Ascomycota, (**E**) Basidiomycota.

**
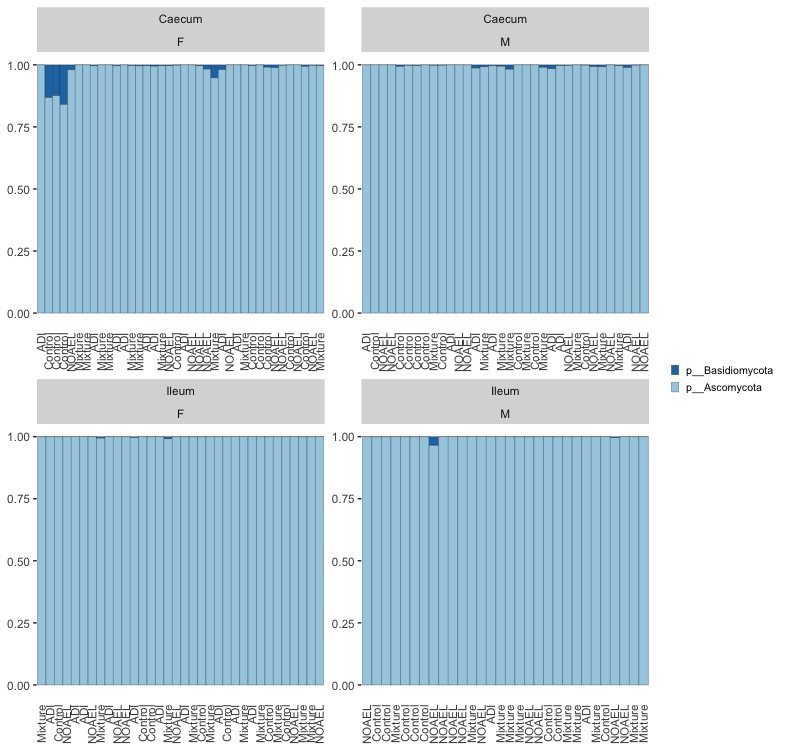
**

**Supplementary Figure 6**. Phylum abundance for the gut fungal analysis in response to treatment with glyphosate at the EU acceptable daily intake (ADI) and no-observed adverse effect level (NOAEL) doses, and a glyphosate, 2,4-D and dicamba mixture (each at the EU ADI). Microbiota composition was determined by ITS2 region sequencing for fungi. Data represents relative abundance of the different ITS2 amplicon sequence variants having their abundance agglomerated at the phylum level.


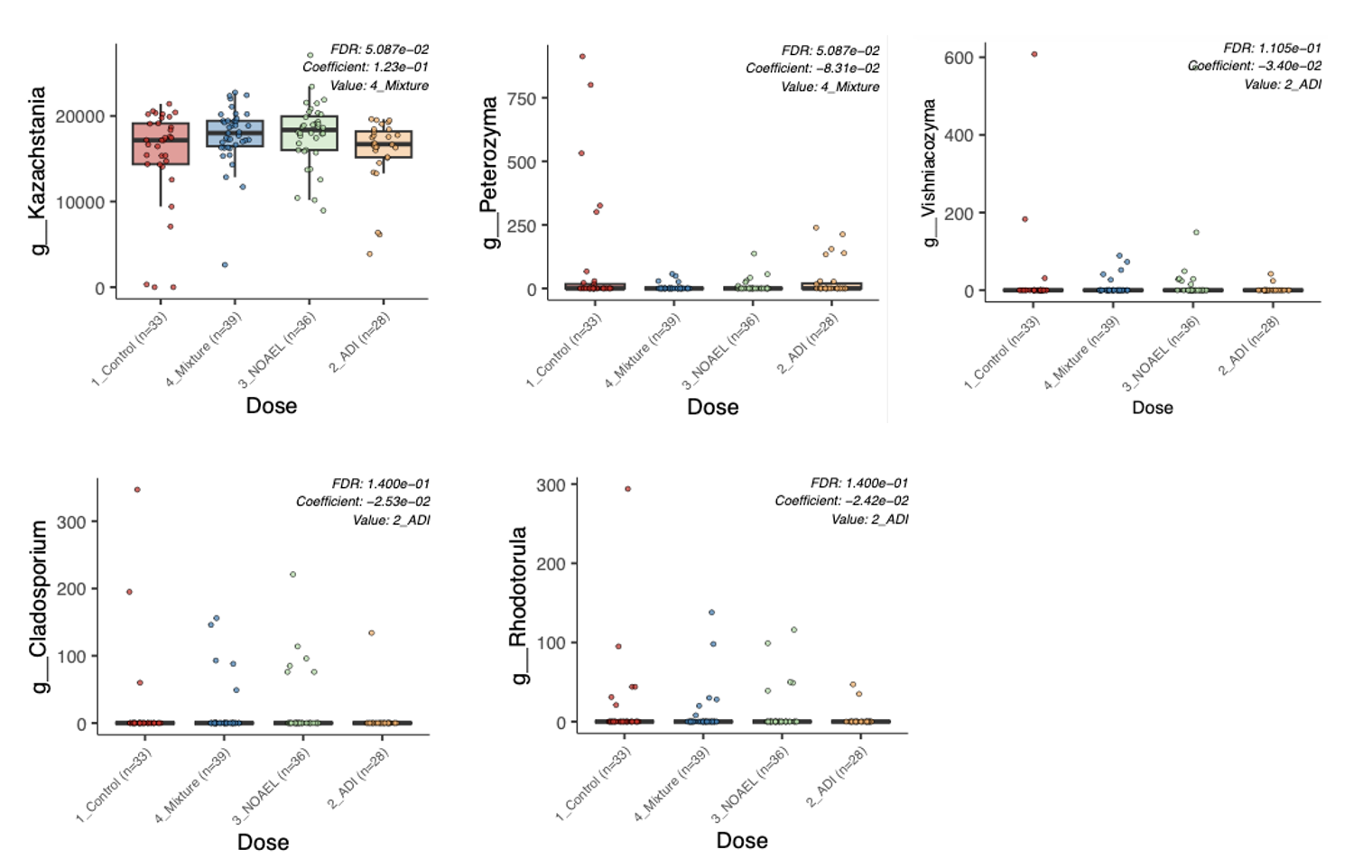


**Supplementary Figure 7 Variation in gut fungal phylum abundance for the different treatment groups.** Microbiota composition was determined by ITS2 region sequencing for fungi in the different animals exposed to glyphosate at the EU acceptable daily intake (ADI) and no-observed adverse effect level (NOAEL) doses, and a glyphosate, 2,4-D and dicamba mixture (each at the EU ADI). Statistical analysis was done using a linear-mixed model with the specialist gut microbiota analysis software Maaslin2. FDR, statistical significance corrected for multiple comparisons. Coefficient, beta value of the linear-mixed model showing changes in abundance in comparison to the control group; Value, group for which the statistical significance is displayed having the most significant variations compared to the control group.


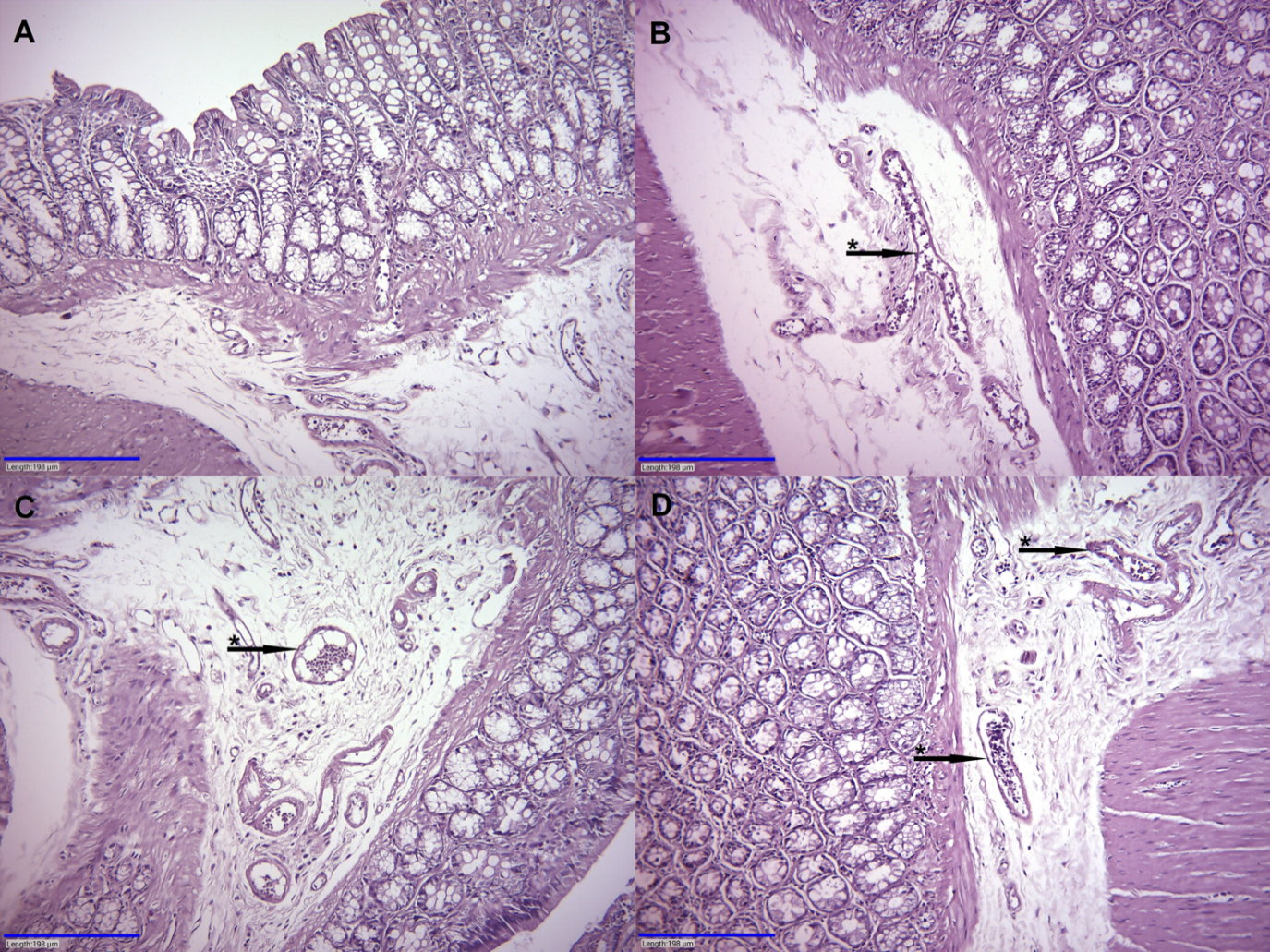


**Supplementary Figure 8. Histological alteration in the large intestine of female rats exposed to glyphosate or its mixture with dicamba and 2,4-D.** Hematoxylin-Eosin staining of large intestine sections from female rats. **A:** control group sample showing normal architecture. **B:** Glyphosate NOAEL dose group sample; dilatated vessels with congestion (black arrow with *). **C:** Glyphosate ADI dose group sample; dilated hyperemic vessel (black arrow with *). **D:** Herbicide mixture group sample; vessels dilatated with vascular congestion (black arrow with *). Magnification: x200.

**
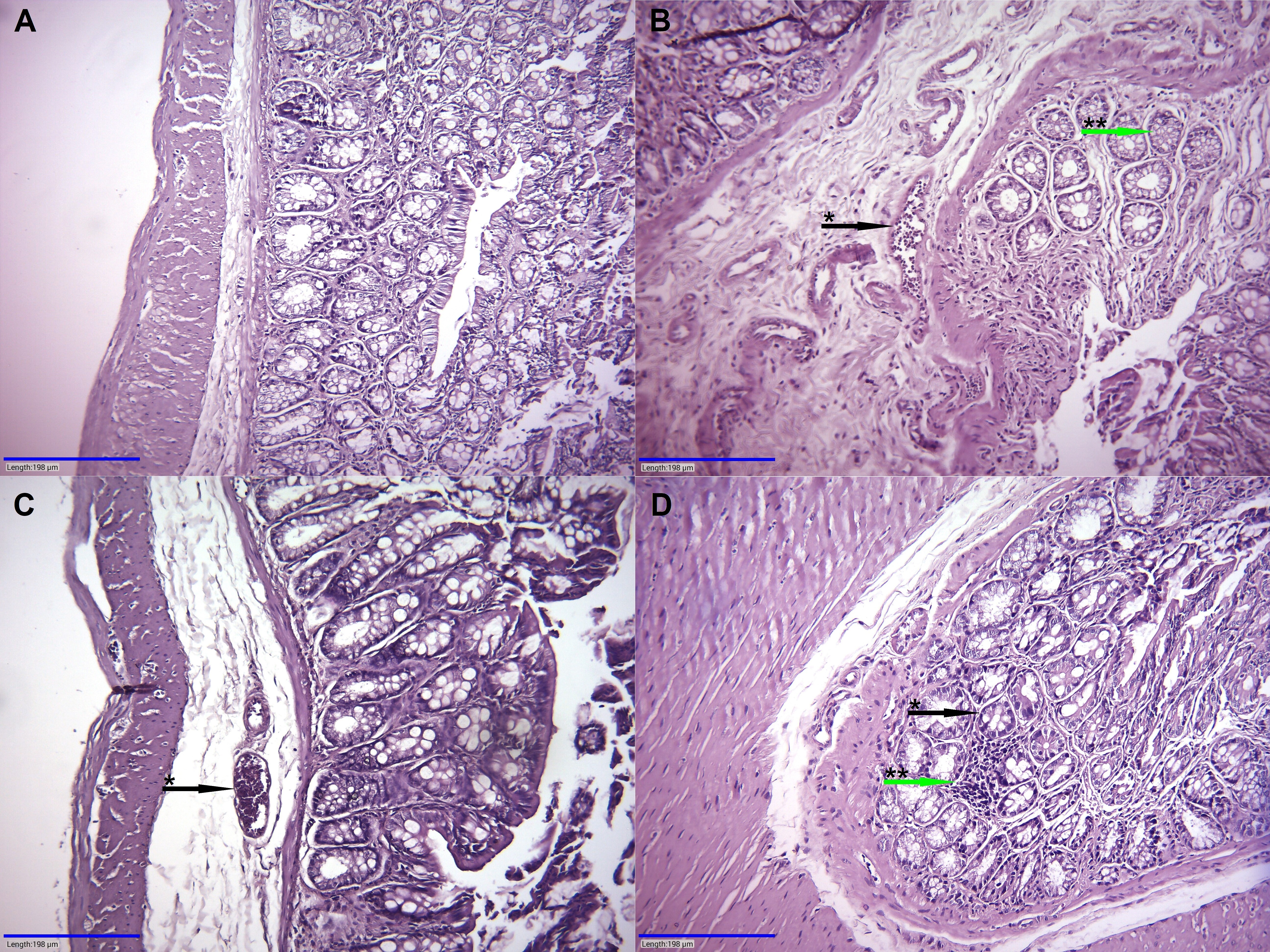
**

**Supplementary Figure 9.** Histological alteration in the large intestine of male rats exposed to glyphosate or its mixture with dicamba and 2,4-D. Hematoxylin-Eosin staining of large intestine sections from male rats. A: control group sample; normal architecture. B: glyphosate NOAEL group sample; hyperemic dilatated vessel (black arrow with *) and glands with microvacuolar cytoplasm (green arrow with **). C: glyphosate ADI group sample; dilatated vessel with congestion (black arrow with *). D: glyphosate, dicamba, 2,4-D mixture group sample; glands with hypertrophic and hyperchromatic nuclei (black arrow with *) and intramural chronic inflammation (green arrow with **). Magnification: x200.


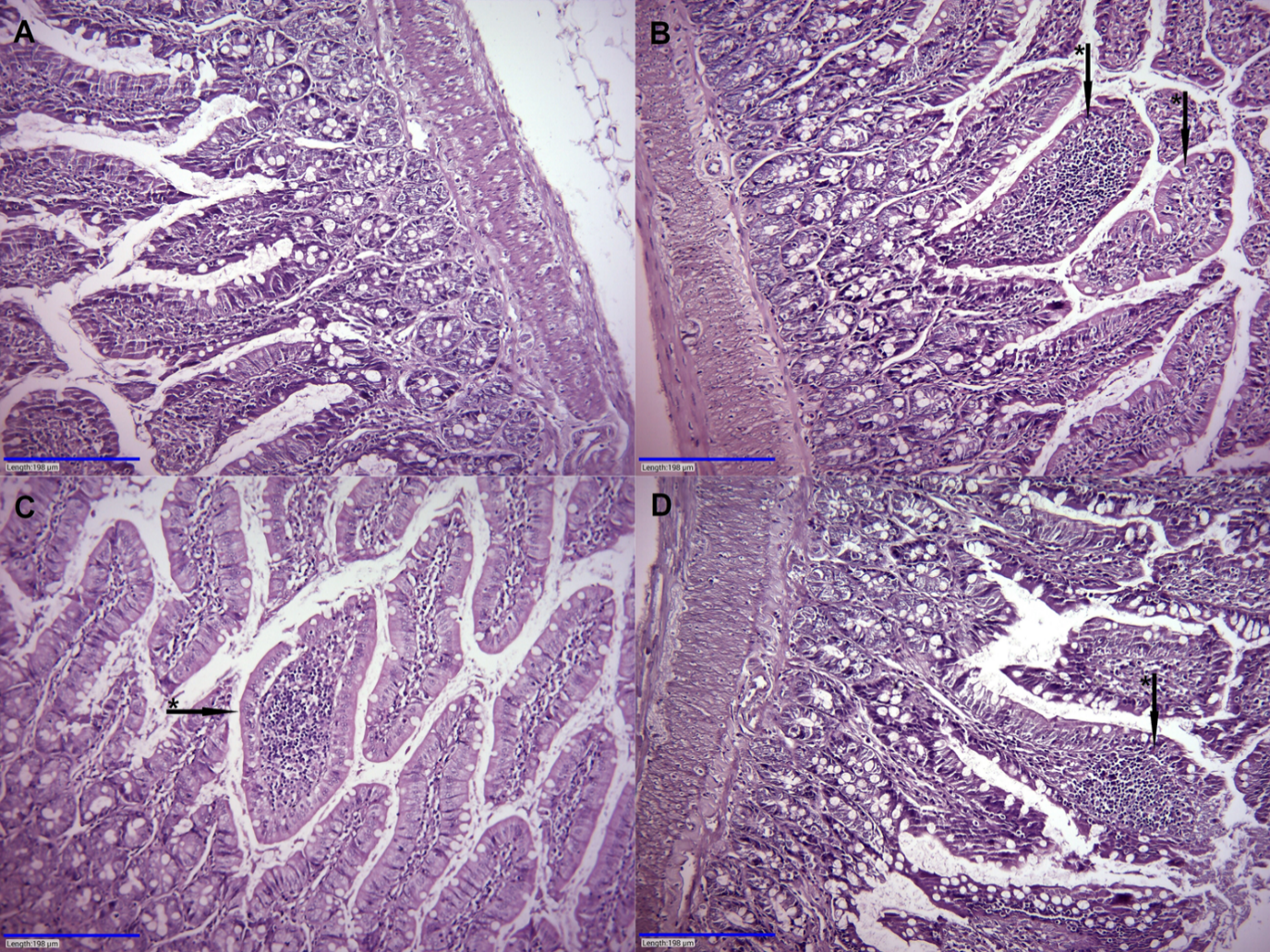


**Supplementary Figure 10. Histological alteration in the ileum of female rats exposed to glyphosate or its mixture with dicamba and 2,4-D.** Hematoxylin-Eosin staining of ileum sections from female rats. **A:** control group sample; normal architecture. **B:** glyphosate NOAEL group sample; chronic inflammation in the villous core (black arrow with *). **C:** glyphosate ADI group sample; villi with chronic inflammation (black arrow with *). **D:** glyphosate, 2,4-D, dicamba mixture group sample; mild chronic inflammation in the villi (black arrow with *). Magnification: x200.

**
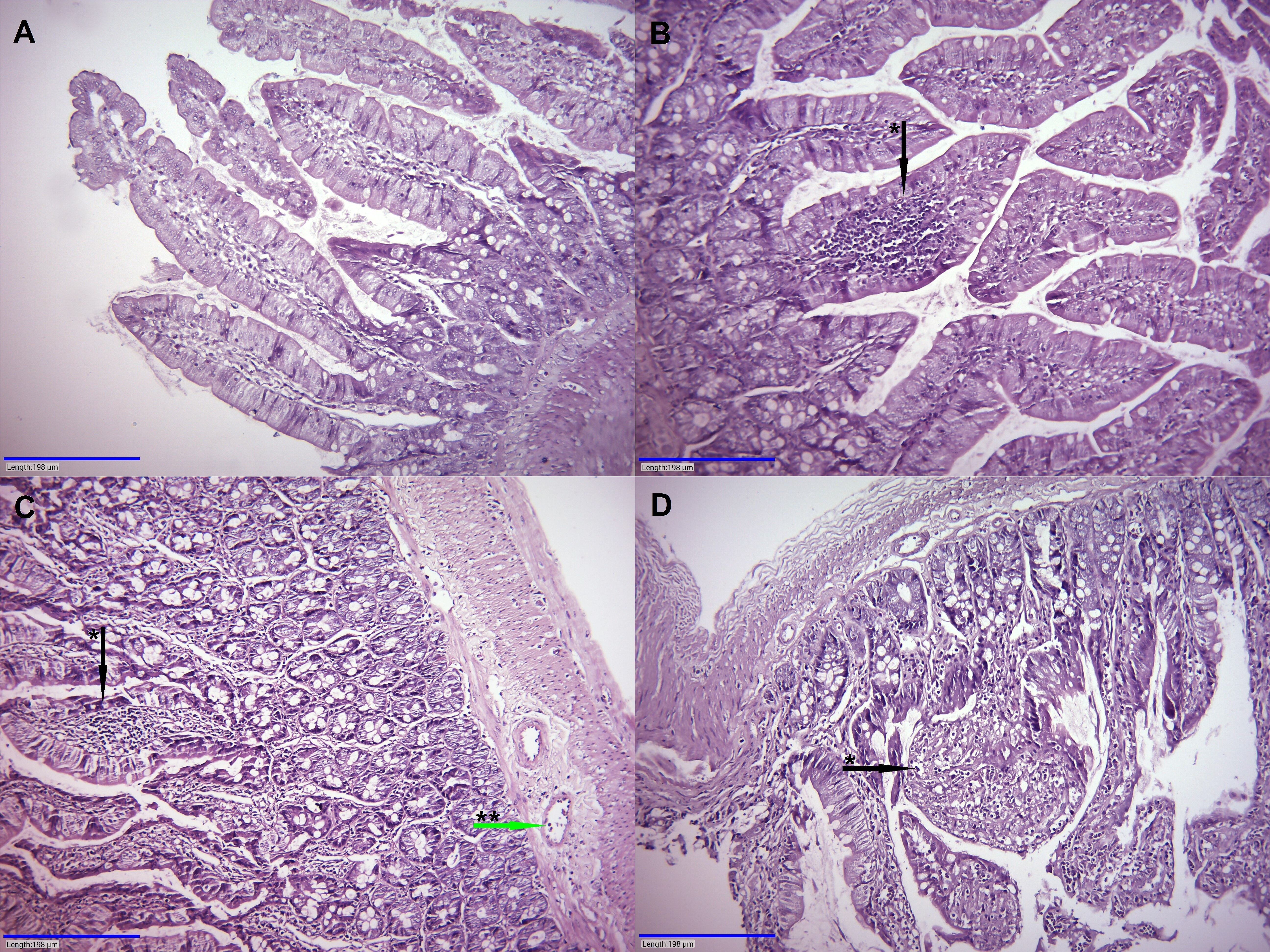
**

**Supplementary Figure 11.** Histological alteration in the ileum of male rats exposed to glyphosate or its mixture with dicamba and 2,4-D. Hematoxylin-Eosin staining of ileum sections from male rats. A: control group sample; normal architecture. B: glyphosate NOAEL group sample; chronic inflammation in the villous core (black arrow with *). C: glyphosate ADI group sample; villi with chronic inflammation (black arrow with *) and dilated slightly hyperemic vessel (green arrow with **). D: glyphosate, 2,4-D, dicamba mixture group sample; dilatated villi with frequent small vessels (black arrow with *). Magnification: x200.
